# Macromolecule‐Driven Supramolecular Polymerization Induced by Crowding Effects

**DOI:** 10.1002/anie.202512216

**Published:** 2025-07-16

**Authors:** Joost J. B. v. d. Tol, Magda M. J. Dekker, Ádám Müller, Puck Springintveld, E. W. Meijer, Ghislaine Vantomme

**Affiliations:** ^1^ Macromolecular and Organic Chemistry Group, Department of Chemical Engineering and Chemistry, Institute for Complex Molecular Systems Eindhoven University of Technology P.O. Box 513 Eindhoven 5600 MB The Netherlands; ^2^ School of Chemistry and RNA Institute the University of New South Wales Sydney 2052 Australia; ^3^ Max Planck Institute for Polymer Research 55128 Mainz Germany

**Keywords:** Crowding, Macromolecules, Sequestration, Supramolecular polymerization

## Abstract

Macromolecular crowding plays a crucial role in biological systems by regulating dynamic processes, yet its effects in fully synthetic environments remain largely unexplored. Here, we systematically investigate how excluded volume effects influence supramolecular polymerizations in organic media. We employ various discotic supramolecular monomers that assemble sequentially into polymers and kinetically‐controlled higher‐order aggregates (HOAs) only in the presence of macromolecular crowders. The phase diagram of the supramolecular assemblies reveals a strong dependence on the macromolecule concentration, size, and polarity, which can be tuned to control polymerization. Remarkably, at high crowder concentrations, large condensed and aligned assemblies were observed in dried samples, suggesting a transition to phase‐separated states. By testing different monomers, macromolecules, and solvents, we establish the general applicability and versatility of macromolecular crowding in guiding supramolecular polymerization. This work provides fundamental insights into assembly processes in crowded environments and opens new avenues for applying macromolecular crowding beyond aqueous systems.

## Graphical abstract

A systematic study is presented that investigates the influence of the excluded volume effect by macromolecules on supramolecular polymerizations in organic media. Upon crowding, the discotic supramolecular monomers sequentially assemble into polymers and higher‐order aggregates (HOAs), affected by the macromolecule's concentration, size and polarity. The proven tunability and general applicability of this entropically driven assembly open new avenues for applying macromolecular crowding beyond aqueous systems.

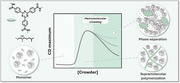

## Introduction

Within the intricate environment of a cell, an astounding diversity of macromolecules, such as disordered proteins, polynucleotides, and polysaccharides, coexist in a limited space.^[^ ^1^, ^2^ ^]^ This densely populated volume gives rise to the macromolecular crowding effect, a phenomenon that controls and maintains the functionality of complex biological systems within the cellular environment.^[^ ^3^, ^4^ ^]^ In physicochemical terms, crowding reduces the accessible volume, modifying the chemical potential and influencing reaction dynamics.^[^ ^3^, ^5^ ^]^ This phenomenon consequently alters biomolecular reaction rates, equilibria, and binding affinities,^[^ ^6^, ^7^, ^8^ ^]^ yet also controls folding processes and stimulates the formation of multi‐subunit complexes.^[^ ^9^, ^10^ ^]^ The crowding effect has been linked to degenerative brain diseases, such as Alzheimer's and Parkinson's, as it affects the fibrillation processes associated with these conditions.^[^ ^11^, ^12^, ^13^, ^14^, ^15^ ^]^

Despite advances in understanding crowding in biological systems,^[^ ^16^, ^17^, ^18^, ^19^, ^20^, ^21^ ^]^ its role in synthetic supramolecular systems remains largely unexplored. Supramolecular polymers are dynamic, reversible assemblies held by noncovalent interactions, making them highly sensitive to environmental factors (e.g., temperature, solvent, and additives)–including the effects of crowding.^[^ ^22^, ^23^ ^]^ Indeed, theoretical studies have shown how crowding‐induced depletion interactions in colloid–supramolecular polymer mixtures can lead to stimuli‐responsive phase behavior.^[^ ^24^ ^]^ Recent literature on this topic has focused mainly on peptide assembly^[^ ^25^, ^26^, ^27^ ^]^ and small molecule crystallization.^[^ ^28^, ^29^, ^30^, ^31^ ^]^ And a very recent study has shown how supramolecular polymers can serve as crowders for other supramolecular polymers.^[^ ^32^ ^]^ However, the excluded volume effect of covalent polymers on supramolecular polymerization has not been systematically studied.^[^ ^33^ ^]^ This lack of knowledge about the crowding phenomenon on supramolecular polymerizations^[^ ^34^, ^35^, ^36^, ^37^, ^38^ ^]^ is the more unexpected, as this effect is active in many processes involving natural supramolecular polymers.^[^ ^39^, ^40^ ^]^

**Figure 1:**
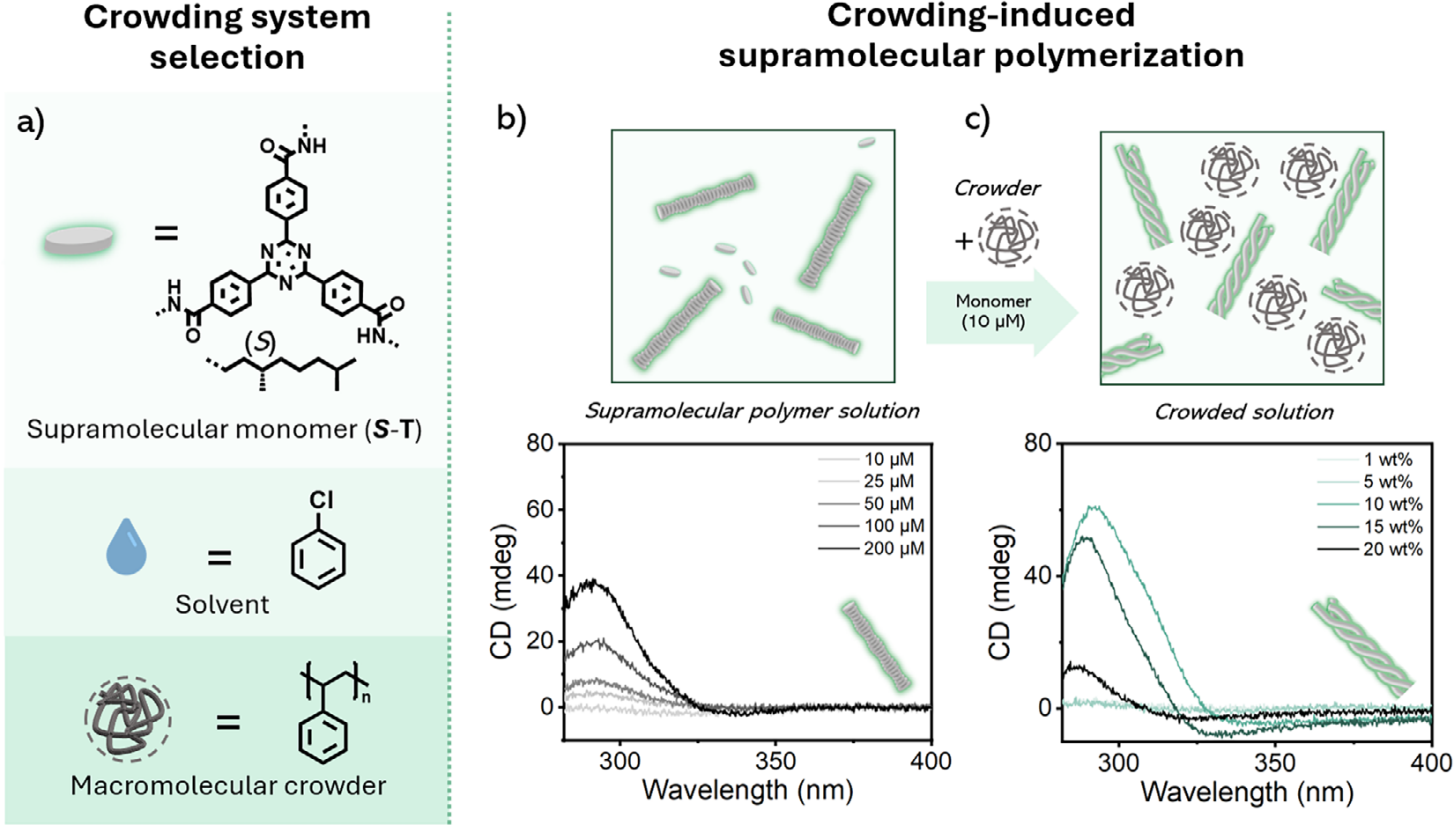
a) Molecular structures of the supramolecular monomer, solvent and macromolecular crowder used for most of the experimental studies. b) Schematic representation and respective CD spectra of ***S***‐**T** at various concentrations in non‐crowded media. c) Schematic representation and respective CD spectra of the crowding‐induced supramolecular polymerization of ***S***‐**T** (10 µM) at various concentrations of polystyrene crowder.

Here, we investigate how macromolecular crowding influences supramolecular polymerization in organic media. We employed discotic chiral supramolecular monomers, such as triazine‐1,3,5‐tribenzenecarboxamide (***S*‐T**),^[^ ^41^ ^]^ which undergo cooperative polymerization and subsequently bundle into higher‐order aggregates (HOAs) in the presence of macromolecular crowders (Figure 1). By carefully selecting solvents and macromolecules with Hansen solubility parameters that match those of the chiral alkylated solubilizing side chains of the monomer,^[^ ^42^ ^]^ we minimize unwanted attractive and repulsive interactions, allowing the non‐covalent forces between monomers to drive the formation of well‐defined one‐dimensional (1D) supramolecular structures. Our results show that crowder concentration, size, and polarity dictate supramolecular polymerization, leading to the formation of phase‐separated states. These findings broaden our understanding of macromolecular crowding in supramolecular chemistry and offer a powerful tool for controlling polymerization pathways in synthetic systems.

## Results and Discussion

### Supramolecular Polymerization in the Presence of Macromolecules

The first step in this study is to choose the right crowder and solvent for the monomer ***S*‐T** in order to accurately address the excluded volume effect. To achieve this, we carefully matched the Hansen solubility parameters (HSP) of the macromolecule‐solvent system with the supramolecular polymer formation.^[^ ^42^ ^]^ This means we examined the solubility of ***S*‐T** in 58 different solvents with known HSPs and selected a macromolecule whose HSP matches that of the supramolecular polymer formation (Figure S5). This step was critical because the introduction of macromolecules alters solution polarity and potentially modifies depletion‐attraction interactions.^[^ ^43^ ^]^ Additionally, the solvent should be able to dissolve the supramolecular monomer at higher temperatures to avoid irreversible kinetic traps and promote reproducible polymerization at room temperature. Based on our previous solvent study,^[^ ^42^ ^]^ we selected polystyrene‐chlorobenzene (PS─CB) as the macromolecule‐solvent system and ***S*‐T** as the supramolecular monomer, ensuring a controlled environment for studying crowding effects (Figures 1a and S5).

We initially probed the supramolecular polymerization of ***S***‐**T** in chlorobenzene without crowder at various concentrations (10, 25, 50, 100, and 200 µM; Figure 1b) using circular dichroism (CD) spectroscopy. The sample preparation involved a slow cooling process (1 °C min^−1^) to avoid kinetic traps and improve reproducibility. As shown in Figure 1b and ***S***‐**T** resides in the monomeric or low assembly state at 10 µM at room temperature, as no Cotton effect was detected. At higher concentrations (>25 µM), supramolecular polymerization occurs, forming chiral 1D assemblies. Corresponding cooling curves imply that ***S***‐**T** undergoes a cooperative assembly process as evidenced by the sharp onset of the CD signal, the so‐called *T*_e_: temperature of elongation (Figure S7).

In contrast, when a macromolecular crowder is introduced (PS, 50 kDa), we observe supramolecular polymerization already at 10 µM ***S*‐T** (Figures 1c and S8–S10). The CD spectrum reveals a pronounced Cotton effect from 10 wt% crowder added, indicating polymerization and bundling into HOAs, as evidenced in Figures 1b,c and S8 by the differences in CD shape, maximum wavelength, and assembly pathway, respectively. We attribute the formation of HOAs to the high effective local concentration of ***S*‐T**, which is triggered by volume exclusion from the polystyrene crowder. This phenomenon can be mimicked in uncrowded environments by increasing the concentration of ***S***‐**T** by 500 to 2000‐fold (Figure S7c). A detailed explanation of the formation of ***S***‐**T** HOAs in crowded environments and the rationale for a kinetically controlled sample preparation are described in section 4.3 of the supporting information.

To generalize this observation, we also examined two other structurally different supramolecular monomers. As shown in Figure S15, a crowding‐induced supramolecular polymerization was also observed for tetraphenyl ethylene (***S***‐**E**) and triphenyl amine (***S***‐**B**) monomers, suggesting the excluded volume effect is a broadly observed phenomenon for hydrogen‐bonded supramolecular polymers.

### The Influence of Crowder Size and Solute Size

**Figure 2:**
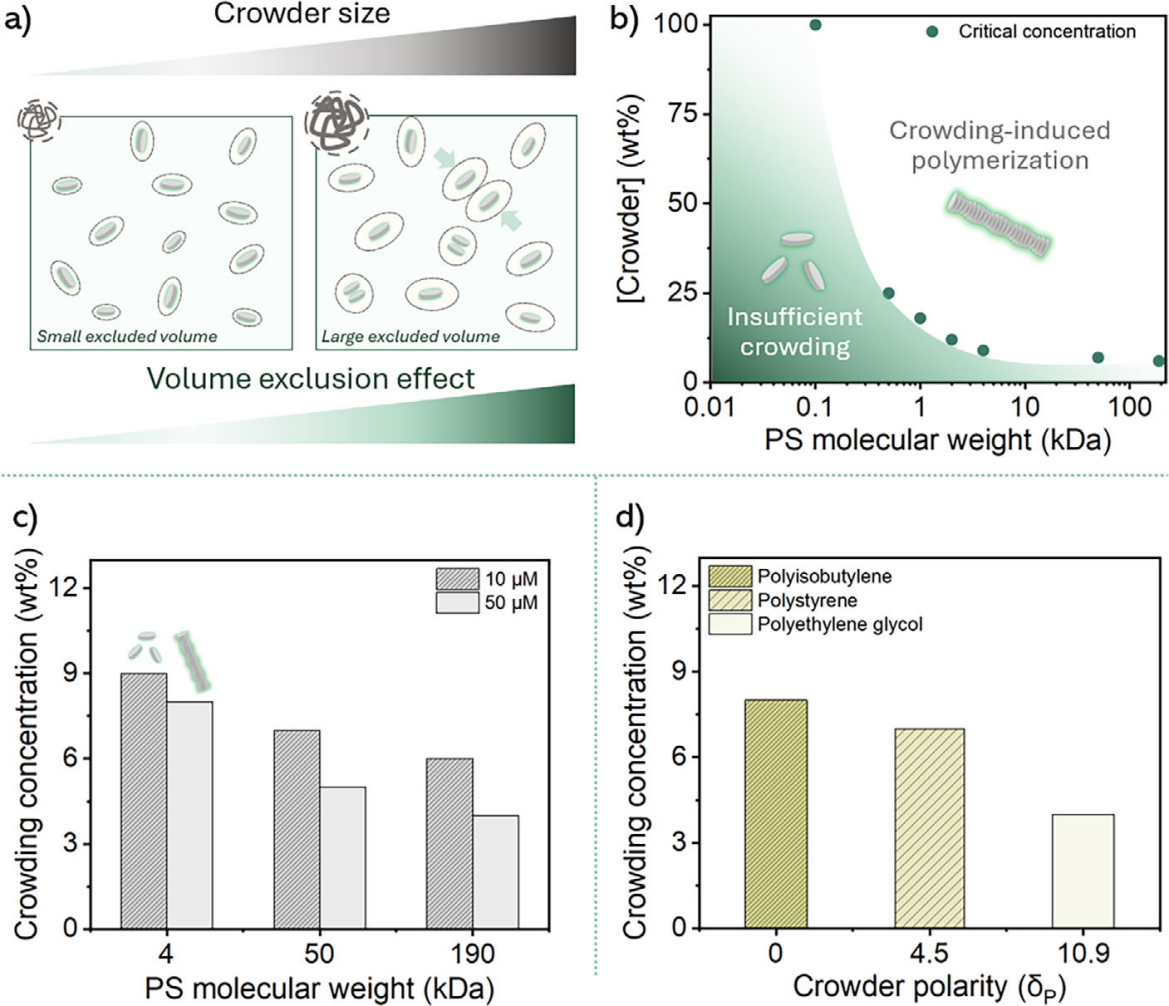
a) Schematic representation of the effect of the crowder size on the volume exclusion effect. b) The observed crowding concentration as a function of the crowder MW for 10 µM ***S***‐**T** solutions. The green region is consistent with no supramolecular polymerization, while the white region is consistent with the formation of ***S***‐**T** supramolecular polymers due to the crowding effect (Figure S12). c) A comparison of the observed crowding concentration between monomeric and polymeric structures of ***S***‐**T**, and thus the solute size, for various crowder sizes. d) The effect of the macromolecule's polarity (*δ*_p_) on the crowding effect for 10 µM ***S***‐**T** solutions with PIB, PS, and PEG as crowders.

The effect of crowder size was explored by varying PS molecular weights (0.5–190 kDa, Table S1). We identified a critical concentration (*c*_cr_) of crowder at which crowding induces supramolecular polymerization (Figures 2b, S11, and S12). The *c*_cr_ decreases steeply with crowder molecular weight (MW) for small crowders (<4 kDa) but plateaus beyond 4 kDa. Intriguingly, this dependence of *c*_cr_ on crowder MW mirrors a typical theoretical scaling of the overlap concentration of macromolecules (c*), suggesting a transition from a dilute to a semi‐dilute regime upon crowding (see discussion below).^[^ ^3^ ^]^

Similarly, the length of ***S***‐**T** supramolecular polymer, which increases with ***S*‐T** concentration, also influences supramolecular polymerization in this crowded environment, as depletion‐attraction interactions become stronger with larger solute size.^[^ ^43^ ^]^ Longer supramolecular polymers experience stronger excluded volume effects, leading to a lower *c*_cr_ compared to monomers. Indeed, crowded 50 µM ***S***‐**T** solutions show lower *c*_cr_ compared to crowded monomeric 10 µM ***S***‐**T** solutions, for three different MWs of PS (Figures 2c and S13). This effect is particularly pronounced at high PS MWs (> 50 kDa), highlighting cooperative depletion interactions between crowding and solute size. These findings highlight the significant parallels that can be drawn between supramolecular polymerizations and their natural analogues.^[^ ^16^, ^44^, ^45^ ^]^

### Effects of Crowder Polarity

Next, we investigated the role of macromolecule polarity using polyisobutylene (PIB), polystyrene (PS), and polyethylene glycol (PEG) as crowders. We selected these macromolecules based on their differences in HSPs (Figure S5) and anticipated different interactions with ***S***‐**T**: polyisobutylene (PIB; *δ*_D _= 18, *δ*_P _= 0, and *δ*_H _= 1), polystyrene (PS; *δ*_D _= 18, *δ*_P _= 4, and *δ*_H _= 3) and polyethylene glycol (PEG; *δ*_D _= 21, *δ*_P _= 11, and *δ*_H _= 13). As shown in Figures 2d and S14 with crowded 10 µM ***S***‐**T** solutions, CD spectra reveal that the propensity for polymerization increases with macromolecule polarity. Indeed, the *c*_cr_ decreases with an increase in crowder polarity, indicating that the thermodynamic activity and effective concentration of ***S***‐**T** increase with increasing crowder polarity. At the same time, repulsive interactions become more prominent as the polarity of the crowder increases, reducing its affinity for the ***S***‐**T** side chains. This result suggests that repulsive interactions enhance depletion effects.

### The Correlation Between Crowder Regime and S‐T Morphology

Inspired by our observations, we investigated the physicochemical principles underlying this phenomenon. Covalent macromolecules typically adopt random coil conformations, exhibiting three concentration regimes: dilute, semi‐dilute, and concentrated. These regimes are defined by two key transition points, the overlap concentration (c*) and the entanglement concentration (*c*_e_), which can be identified through changes in specific viscosity. Notable changes in specific viscosity with increasing macromolecule concentration mark these transitions.

**Figure 3:**
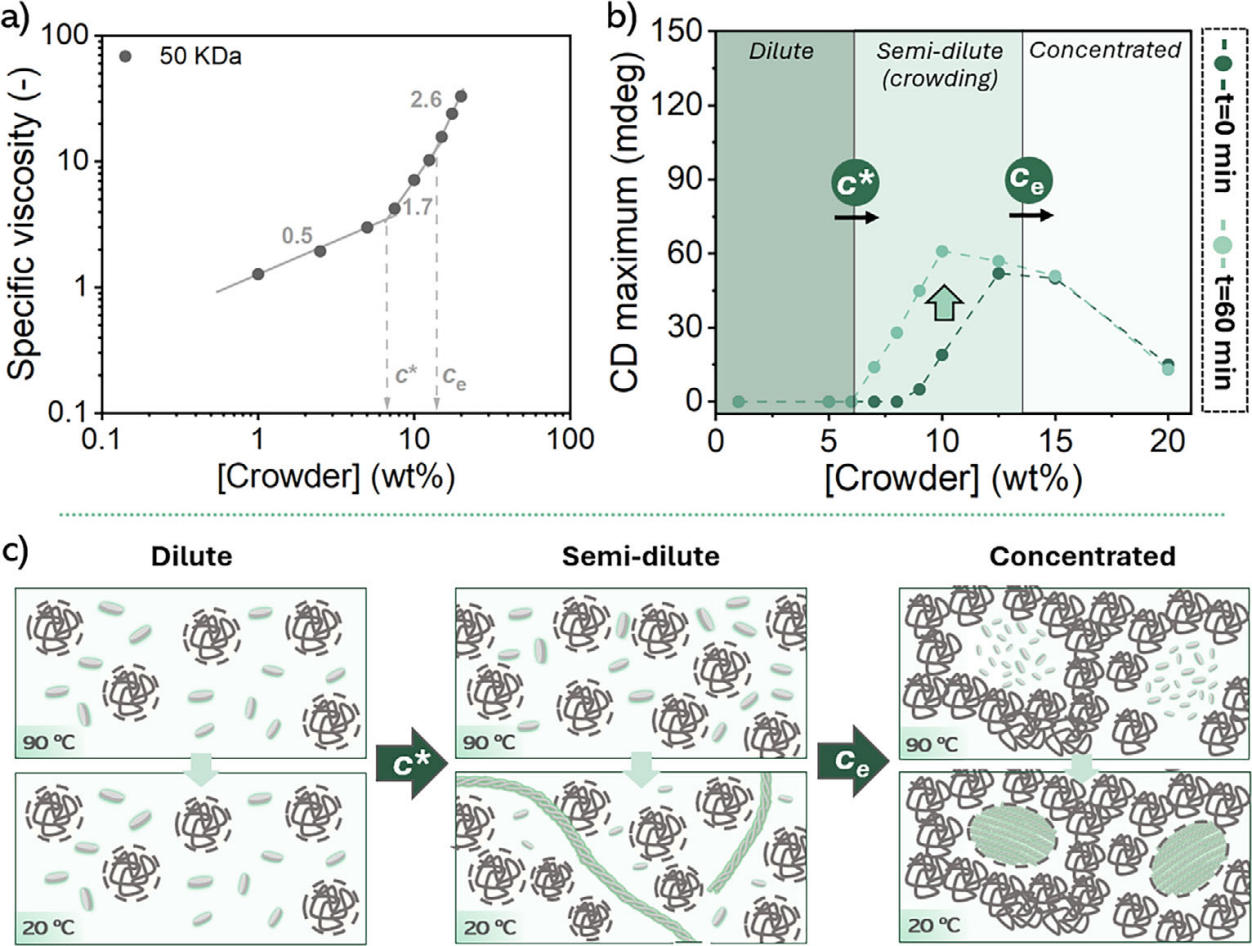
a) The specific viscosity of crowder in chlorobenzene plotted as a function of crowder concentration. The c* and *c*_e_ can be extracted from the inflection points. b) The maximum CD signal of crowded 10 µM ***S***‐**T** solutions in chlorobenzene (at *t* = 0 and 60 min) plotted as a function of crowder concentration. The highlighted areas represent the distinct crowder concentration regimes, which are defined by the experimentally determined c* and *c_e_*. In all experiments, a PS crowder is used with a MW of 50 kDa. c) Schematic representation of the three distinct crowder concentration regimes (dilute, semi‐dilute, and concentrated), illustrating the proposed supramolecular morphologies at 90 °C and upon cooling to 20 °C.

As shown in Figure 3a, an inflection in the slope of the specific viscosity is observed at 6.6 and 13.5 wt% PS (MW = 50 kDa), marking c* and *c*_e_, respectively. Notably, c* closely matches *c*_cr_ (7 wt%), indicating that overlapping PS coils induce crowding‐driven supramolecular polymerization of ***S*‐T**. This trend holds across different molecular weights (DPs of 1, 5, 10, 20, 40, 500, 1900, Figures S12, S16, and S17), confirming that *c*_cr_ represents the boundary between the dilute (non‐crowded) and semi‐dilute (crowded) regimes in 10 µM ***S***‐**T** solutions.^[^ ^3^, ^46^ ^]^ It is important to note that this crowding‐driven supramolecular polymerization is not merely due to increased viscosity slowing diffusion; rather, it results from excluded volume effects that reduce the available space for supramolecular components, thereby increasing their effective concentration.

To further explore these effects, and given that crowding introduces kinetic control, we analyzed the CD signal at two time‐points (*t* = 0 and 60 min) as a function of crowder concentration (Figure 3b). Above c*, the increasing CD signal over time indicates enhanced depletion‐attraction interactions, driving the growth and bundling of supramolecular polymers into higher‐order assemblies (HOAs). Additionally, a rise in effective ***S*‐T** concentration correlates with higher critical assembly temperature (*T_c_*) and a shorter stabilization time for the Cotton effect (Figures S18 and S19).

**Figure 4:**
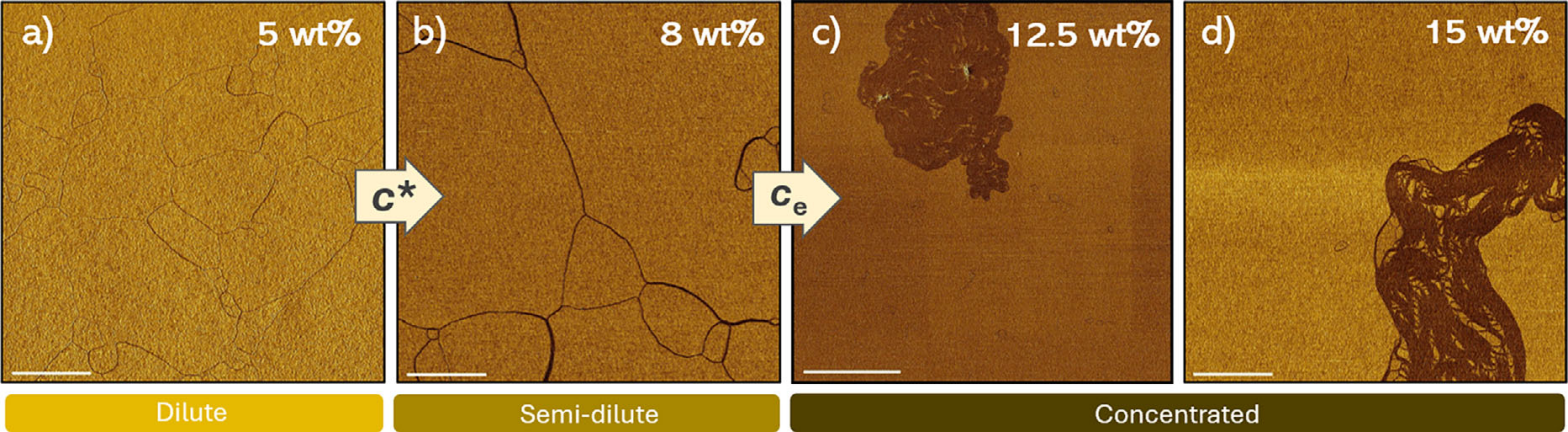
a)–d) 10x10 µm AFM phase images of spincoated samples of 10 µM chlorobenzene ***S***‐**T** solutions with a) 5, b) 8, c) 12.5, and d) 15 wt% of PS crowder (50 kDa). All inset scalebars represent 2 µm.

At crowder concentrations exceeding *c*_e_, supramolecular polymer growth reaches a limit, as indicated by the decreasing CD signal (Figure 3b). Atomic force microscopy (AFM) analysis shows the persistence of large ***S*‐T** assemblies, ruling out crowding‐induced depolymerization as a cause for the CD decrease (Figures 4 and S20). Instead, this may result from a change of assembly pathway at high crowding levels, leading to HOAs with opposite helicity^[^ ^47^, ^48^ ^]^ and consequently reduced CD intensity. The effect is amplified by polar crowders (e.g., PEG at 15 wt%), likely due to additional repulsive interactions and reduced crowder affinity for ***S*‐T** side chains (Figure S14c). The stability of the CD signal over time in highly crowded conditions (c > *c*_e_) suggests that ***S*‐T** assemblies become confined with limited diffusion, a behavior reminiscent of reaction‐ and diffusion‐limited crowding effects seen in biological systems (Figure 3c).^[^ ^49^ ^]^

To further support the spectroscopic data, AFM phase‐imaging was conducted on spin‐coated ***S***‐**T** samples prepared from crowded solutions (5, 8, 12.5, and 15 wt% PS), as depicted in Figures 4 and S20. Unlike the spectroscopic results, a network of thin fibers was detected at 5 wt% PS, likely due to concentration gradients during drying (Figure 4a). At PS crowder concentrations ≥8 wt%, increasing depletion–attraction interactions sequentially lead to an entropy‐driven bundling of ***S*‐T** into thick fibers and formation of large, condensed, and aligned assemblies at 12.5–15 wt% PS (Figure 4b,d). The latter phase likely corresponds to the confinement of HOAs into phase‐separated states. Confocal microscopy further confirmed the formation of phase‐separated structures in crowded 20 µM ***S*‐E** toluene solutions at crowder concentrations ≥10 wt% (Figure S21).

To elucidate the difference in molecular packing between supramolecular polymers, HOAs, and phase‐separated HOAs, FT‐IR spectroscopy was performed on crowded (500 µM ***S*‐B**) and non‐crowded solutions, as 10 µM ***S***‐**T** crowded solutions did not provide a sufficient signal to noise ratio. While bundling into HOAs did not alter N─H stretch frequencies (3275 cm⁻¹; Figure S23), further increasing crowder concentrations (20–30 wt%) caused a shift to 3302 cm⁻¹ (Figure S24d,e). This suggests weakened hydrogen bonding or N─H group reorientation, potentially indicating changes in supramolecular polymer alignment.^[^ ^50^ ^]^ This hypothesis is supported by the fact that the shift occurs gradually over time, representing a relatively slow process such as fiber alignment, which accelerates as crowder concentrations increase.^[^ ^51^ ^]^ Time‐dependent AFM imaging further supports a gradual transition into condensed, aligned phases as crowder concentrations increase, resembling fiber alignment in LLPS systems (Figure S24e,f).^[^ ^49^ ^]^

### Disfavoring Bundling Pathway Using Randomized Side Chains

**Figure 5:**
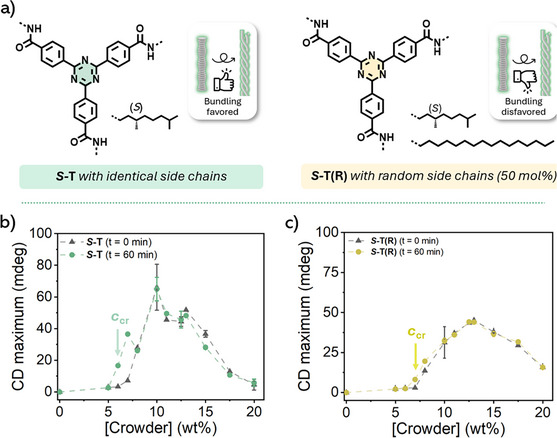
a) Molecular structures of ***S***‐**T** and ***S***‐**T(R)**, equipped with identical and randomized side chains, respectively. ***S***‐**T(R)** is functionalized with equimolar amounts of chiral C_8_‐ and achiral C_17_‐side chains, giving a statistical mixture of four distinct molecules. The maximum CD signal as function of the crowder concentration for crowded 10 µM b) ***S***‐**T** and c) ***S***‐**T(R)** solutions in chlorobenzene. In all experiments, PS with a MW of 190 kDa is used as crowder.

The above study showed that depletion‐attraction interactions alter the assembly pathways of the supramolecular polymer ***S*‐T**, leading to HOA kinetic traps and reduced reproducibility. These effects are particularly pronounced at high crowder concentrations (c > *c_e_*), with high molecular weight crowders (MW PS ≥ 50 kDa) or in the presence of repulsive interactions. To mitigate these effects, we aimed to simplify the pathway complexity of ***S*‐T** through side‐chain engineering, disfavoring the uncontrolled formation of HOAs by introducing randomized side chains instead of uniform ones.^[^ ^52^ ^]^ Random‐length side chains are expected to reduce secondary nucleation on the fibers, thereby limiting bundling and HOA formation. Hereto, we compared the supramolecular polymerization of **S‐T**, which carries identical chiral C_8_‐side chains, with ***S*‐T(R)**, a variant functionalized with an equimolar mixture of chiral C_8_‐ and achiral C_17_‐side chains, in a crowded environment (Figure 5a). The introduction of random side chains results in a statistical mixture of four distinct ***S*‐T(R)** molecules (Supporting Information, Section 2).

After confirming that ***S*‐T(R)** assembles similarly to ***S*‐T** (Figure S25), we investigated the effect of randomized side chains in crowded media containing high MW PS (190 kDa). As shown in Figure 5b,c, reproducibility is improved for ***S***‐**T(R)** as both the variations in the maximum CD and the corresponding standard deviations are significantly smaller than for ***S***‐**T**. Furthermore, the onset of the crowding regime for ***S*‐T(R)** is shifted to higher crowder concentrations, from 6 to 7 wt% for 190 kDa PS (Figure 5c) and from 10 to 12 wt% for 50 kDa PS (Figure S27) compared to ***S*‐T**. These results suggest that side‐chain randomization delays and disfavors the bundling pathway, offering a practical strategy to mitigate kinetic traps, enhance reproducibility, and facilitate controlled sample preparation. While the overall effect is clear, the precise contribution of each individual analogue in the statistical mixture remains unclear.

### Steering Supramolecular Polymerizations Using Macromolecules

Thus far, we have explored macromolecules as inert crowders, which induce repulsive interactions that promote supramolecular polymerization. Building on this, we designed a system where macromolecules play the opposite role, functioning as sequestrators.^[^ ^53^ ^]^ In this case, the interaction between the sequestrating macromolecule and the supramolecular monomer must be strong enough to stabilize the monomer and effectively prevent supramolecular polymerization. In the ***S***‐**T**/PEG system, for example, interaction between ***S***‐**T** monomers is favored over interaction between ***S***‐**T** and PEG, resulting in crowding rather than sequestration.

For this purpose, we selected triphenylamine ***S*‐A**, a supramolecular monomer with weaker intermolecular interactions than ***S***‐**T**. Based on its HSPs,^[^ ^42^ ^]^ we identified polymethyl methacrylate (PMMA) as an effective sequestrating macromolecule, acting as a good “solvent” where ***S*‐A** remains molecularly dissolved (Figure S6). Meanwhile, polystyrene (PS) was used as a crowding macromolecule, with toluene as the solvent, both of which promote ***S*‐A** supramolecular polymerization.

**Figure 6:**
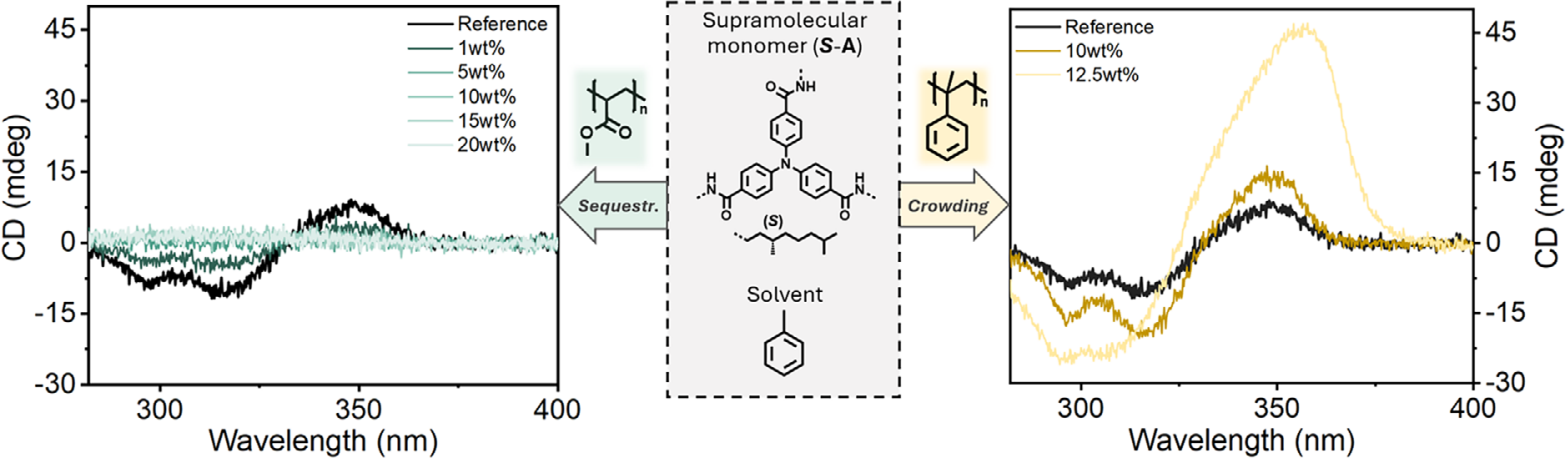
CD spectra of ***S***‐**A** (250 µM) in the presence of sequestrator (PMMA, left) and crowder (PS, right). In both cases, toluene is used as a solvent to promote supramolecular polymerization.

Starting from a 240 µM ***S*‐A** solution in toluene, PMMA sequestrates ***S*‐A** at just 1 wt%, with full sequestration occurring at 5 wt% PMMA (Figure 6, left), as evidenced by the disappearance of the Cotton effect and a red‐shift in UV absorbance from the polymeric to the molecularly dissolved state (Figure S28).^[^ ^54^ ^]^ Interestingly, further increasing PMMA concentration (10–20 wt%) does not induce crowding effects, likely due to strong attractive interactions between ***S*‐A** and PMMA, with PMMA acting as a hydrogen‐bond acceptor. Conversely, when PS replaces PMMA, a significant increase in CD signal at 10–12.5 wt% is observed (Figure 6, right), confirming that PS induces supramolecular polymerization through crowding effects.

These findings demonstrate the ability to rationally design supramolecular polymer‐macromolecule systems that exert precise control over supramolecular polymerization. They also highlight that macromolecular crowding is a general phenomenon in supramolecular polymerization in organic media, emphasizing its importance in controlling self‐assembly pathways in complex systems.

### Effect of Macromolecular Matrix in Bulk Formulation

Finally, we tested our HSP approach on the influence of polymer matrices on the supramolecular assembly of perylene bisimide (PDI) *J*‐aggregates, as exemplified in a recent study by the group of Würthner.^[^ ^33^ ^]^ In their work, PDI dyes formed well‐defined *J*‐aggregates in polystyrene (PS), while kinetically trapped monomers, larger three‐dimensional crystallites, and phase‐separated structures were formed in PMMA and poly(styrene‐butadiene‐styrene) (SBS) matrices. These results can be interpreted through the lens of solubility parameter matching and macromolecular crowding. Specifically, the HSP of PS matches with a typical *J*‐aggregate solubility region for supramolecular monomers, such as PDI and porphyrin,^[^ ^37^ ^]^ while both PMMA and SBS align more closely with the solubility region of HOA, favoring kinetic traps, phase separation, and consequent crystallization. Thus, matching the HSP of macromolecular hosts with functional supramolecular monomers is crucial to control their self‐assembly pathways and optimize optoelectronic performance in solid‐state devices.

## Conclusions

This study demonstrates how macromolecular crowding serves as a tool to steer supramolecular polymerization in organic media, revealing critical insights into excluded volume effects, phase separation, and pathway complexity. Through a systematic approach, we isolated the impact of excluded volume effects from additional attractive or repulsive interactions reported in other studies,^[^ ^16^, ^27^, ^30^, ^31^ ^]^ providing a clearer understanding of how macromolecules can influence self‐assembly dynamics. By employing spectroscopic, microscopic, and rheological analyses, we established a direct correlation between ***S*‐T** morphological transitions and distinct crowder concentration regimes. At high crowder concentrations, we observed the emergence of condensed, aligned supramolecular structures. This study also highlights the dual role of macromolecules, functioning as either crowders or sequestrators, depending on their affinity for supramolecular monomers. By modulating macromolecular polarity and interactions, we demonstrated a strategy to control supramolecular polymerization pathways, providing an effective approach to improve reproducibility, mitigate kinetic traps, and delay undesired bundling events.

The presented findings underscore the potential of macromolecular crowding as a design principle in supramolecular chemistry, expanding its relevance beyond aqueous systems to organic media. This crowding‐induced supramolecular polymerization is also relevant to industrial formulations, where thickeners create similarly crowded environments that can influence self‐assembly, gelation, and material stability. The complexity of interactions between crowding, solvation, sequestration, and supramolecular assembly highlights striking parallels between natural and synthetic self‐assembling systems. This study opens new avenues for rational formulation design, enabling greater control over supramolecular polymerization and hierarchical material assembly, paving the way for advanced functional materials.

## Supporting Information

Materials and methods; synthetic procedures; sample preparation; Hansen solubility spaces; supporting CD, UV and FTIR spectra; AFM and confocal images; supporting viscosity data.

## Author Contributions

The research was designed by J.v.d.T., E.W.M. and G.V., and the experiments are performed by J.v.d.T., M.D., A.M., and P.S. the manuscript was written through contributions of all authors. All authors have given approval to the final version of the manuscript.

## Conflict of Interests

The authors declare no conflict of interest.

## Supporting information



Supporting Information

## Data Availability

The data that support the findings of this study are available in the Supporting Information of this article.
