# Supplementary material for: Macromolecule‐Driven Supramolecular Polymerization Induced by Crowding Effects
Source: Angew Chem Int Ed Engl. 2025 Jul 16;64(36):e202512216. doi: 10.1002/anie.202512216 (PMC12402893; doi:10.1002/anie.202512216)
Supplement: Supplementary file 1 — Supporting Information [file ANIE-64-e202512216-s001.pdf]

Supporting information

Macromolecule-Driven Supramolecular Polymerization Induced by Crowding Effects

Joost J. B. v. d. Tol[1], Magda M. J. Dekker[1], Ádám Müller[1], Puck Springintveld[1], E. W. Meijer[1,2,3], and Ghislaine Vantomme[1]

[1] *Institute for Complex Molecular Systems and Laboratory of Macromolecular and Organic Chemistry, Eindhoven University of Technology, P.O. Box 513, 5600 MB Eindhoven, The Netherlands.*
[2] *School of Chemistry and RNA Institute the University of New South Wales, 2052 Sydney, Australia.*
[3] *Max Planck Institute for Polymer Research, 55128, Germany.*

Table of Contents

1. Materials and methods 2
2. Synthesis of supramolecular monomers 3
3. Macromolecule-solvent system selection 7
4. Thermodynamic versus kinetically controlled CD measurements 8
5. CD data of supramolecular polymerizations in crowded media 12
6. The correlation between crowder regimes and evolution of CD 14
7. Morphology of crowded solutions of ***S*-T** & ***S*-E** 17
8. CD and infrared spectroscopy of highly concentrated solutions of ***S*-B**. 18
9. Spectroscopic characterization of ***S*-T(R)** in uncrowded and crowded media 20
10. Sequestration versus crowding of supramolecular building block ***S*-A** 21
11. References 22
Appendix A 23

1. Materials and methods

S1.1 Materials

All reagents and polymers were purchased from commercial resources and used without further purification. The supramolecular building blocks ***S*-T**, ***S*-A** and ***S*-E** were obtained from previously prepared batches.[1–4] The supramolecular building block ***S*-B** and chiral tail (*S*)-3,7-dimethyloctan-1-amine were synthesized following reported procedures.[1,5] All solvents were purchased from commercial resources, purified by passing through a basic aluminium plug and dried over 4 Å molsieves for at least 24 hours before use. Deuterated solvents were obtained from Cambridge Isotopes Laboratories. Oven-dried glassware (120 °C) was used for all reactions carried out under argon atmosphere. Reactions were followed by thin-layer chromatography (TLC) using Merck's 60-F_{254} silica gel plates and when necessary visualized by potassium permanganate ($KMnO_4$) stain. Column chromatography was performed on Screening Devices silica gel (40-63 µm, 60 Å).

Table S1. Characteristics of macromolecules used in this study.

Entry[a]	Polymer type[b]	Mw[c] [g/mol]	Mn[d]	PDI[e] [-]
**PS(0.5)**	Polystyrene	520	470	1.11
**PS(1)**	Polystyrene	1110	1020	1.10
**PS(2)**	Polystyrene	1890	1790	1.06
**PS(4)**	Polystyrene	4130	4110	1.01
**PS(50)**	Polystyrene	50000	-	-
**PS(190)**	Polystyrene	192000	-	-
**PEG(20)**	Polyethylene glycol	-	20000	-
**PIB(500)**	Polyisobutylene	500000	200000	2.5
**PMMA(15)**	Polymethyl methacrylate	15000	-	-

[a] Polymer abbreviations and [b] polymer types that are employed in this study. [c] Weight average molecular weight (Mw), [d] number average molecular weight (Mn) and [e] polydispersity index (PDI) of the polymers utilized as reported by the commercial source.

S1.2 Methods

NMRs spectra were recorded using a Varian Mercury Vx 400 MHz and Bruker ASCEND spectrometer (^{1}H-NMR using 400 MHz and ^{13}C-NMR using 100 MHz). Proton and carbon chemical shifts are reported in ppm (δ) downfield from tetramethylsilane (TMS) using the deuterated solvent resonance frequency as internal standard. Peak multiplicities are abbreviated as s: singlet; d: doublet; t: triplet; q: quartet; p: pentet; m: multiplet; dd: double doublet; dt: double triplet and dq: double quartet.

Matrix assisted laser absorption/ionization mass time of flight (MALDI-TOF) measurements were performed on a Bruker Autoflex Speed using α-cyano-4-hydroxycinnamic acid (CHCA) and *trans*-2-[3-(4-*tert*-butylphenyl)-2-methyl-2-propenylidene]malononitrile (DCBT) as matrices.

UV/Vis and circular dichroism (CD) measurements were performed on a JASCO J-815 CD spectrometer, equipped with either a JASCO Peltier MPTC-490S temperature controller (temperature range of 278 – 373 K) or a JASCO Peltier PFD-425S/15 (temperature range of 263 – 383 K), and a Jasco FMO-427S/15 emission monochromator. Hellma Quartz Suprasil cuvettes with an optical pathlength of 0.1 cm were used for all spectroscopic measurements.

Fourier-Transform Infrared (FT-IR) transmission spectra were obtained using a Shimadzu IR-Tracer 100 Fourier-Transform Infrared spectrometer coupled to a TGS detector. A measurement cell equipped with CaF cell windows and a 0.5 mm spacer was used. The spectra were recorded with 4 cm^{-1} resolution and averaged over 100 scans for the samples.

Atomic Force Microscopy (AFM) studies were performed using a Cypher Environmental Scanner (ES) equipped with a closed cell and an active heating and cooling stage. Silicon probes (AC160) with a tip height of 10–15 µm, radius of 7 nm, frequency of 300 kHz and a force constant of 26 N/m were used to record images in tapping-mode (phase<90). A scan rate of 2-3 Hz and a resolution of 1024x1024 pixels were used. Samples were prepared by spincoating 20 µL of crowded solutions with 1500 rpm for 30 seconds onto freshly cleaved 1x1 cm^2 sized mica followed by overnight drying in air. Contrast of the images was further enhanced using first order planefit and flattening using Gwyddion v2.60.

Viscosity measurements were performed on a Lovis 2000 M/ME rolling ball viscometer equipped with a 1.8 mm capillary using a gold coated ball to prevent interactions with the solute. Prior to the measurements, the viscometer is calibrated according to ISO 17025 with a standard (APS6). All dynamic viscosity measurements were performed at 20 °C by measuring either the long- or short-distance time at an angle of 45°. The reference dynamic viscosity of chlorobenzene was acquired from literature. Subsequent specific viscosities were obtained using the dynamic viscosity of the sample and chlorobenzene.

2. Synthesis of supramolecular monomers

S2.1 Synthesis of 4,4',4"-(1,3,5-triazine-2,4,6-triyl)tris(N-((S)-3,7-dimethyloctyl) benzamide) with randomized side chains [*S*-T(R)]

4,4’,4’’-(1,3,5-triazine-2,4,6-triyl)tribenzoic acid (160 mg, 0.36 mmol, 1 eq.) was suspended in dry DMF (5 mL) under argon atmosphere. HATU (506 mg, 1.34 mmol, 3.7 eq.) and DIPEA (226 mg, 1.74 mmol, 4.8 eq.) were added, upon which the mixture was stirred for 10 minutes. (*S*)-3,7-dimethyloctan-1-amine (114 mg, 0.72 mmol, 2.0 eq.) and hexadecyl amine (175 mg, 0.72 mmol, 2.0 eq.) were added and the mixture was stirred at 50°C for 12 hours. The yellow solution was left to cool down and diluted with chloroform (100 mL). The solution was washed with 1M HCL (250 mL), water (100 mL) and brine (100 mL). The organic layer was collected, dried over $MgSO_4$, and concentrated *in vacuo*. The product was purified by silica gel column chromatography (EtOAc/$CHCl_3$ 3:17). Precipitation from DCM into MeOH afforded ***S*-T(R)** as a white solid in 37% yield (131 mg).

^{1}H NMR (400 MHz, Chloroform-d_1 and 3 vol% TFA- d_1): δ [ppm] = 8.54 (d, J = 8.3 Hz, 6H), 7.77 (d, J = 8.3 Hz, 6H), 7.02 (br, 3H), 3.66 – 3.47 (m, 6H), 1.79 – 1.65 (m, 4.5H), 1.62 – 1.48 (m, 4.5H), 1.48 – 1.11 (m, 48H), 0.99 (d, J = 6.3 Hz, 4.5H), 0.91 – 0.84 (m, 13.5H). ^{13}C NMR (101 MHz, Chloroform-d_1 and 3 vol% TFA-d_1): δ [ppm] = 170.55, 169.86, 138.85, 136.24, 129.33, 127.37, 118.65, 115.81, 112.97, 110.13, 41.47, 39.67, 39.23, 37.10, 36.13, 31.98, 30.95, 29.75, 29.71, 29.64, 29.58, 29.41, 29.29, 29.10, 28.01, 26.98, 24.69, 22.73, 22.67, 22.57, 19.37, 14.12. MALDI-TOF-MS found: 859.6, 943.7, 1027.8, 1111.9 m/z (calculated $[M+H]^+$: 858.6, 942.7, 1026.8, 1110.9).

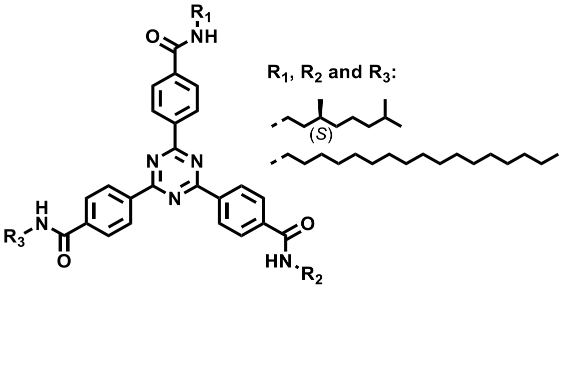

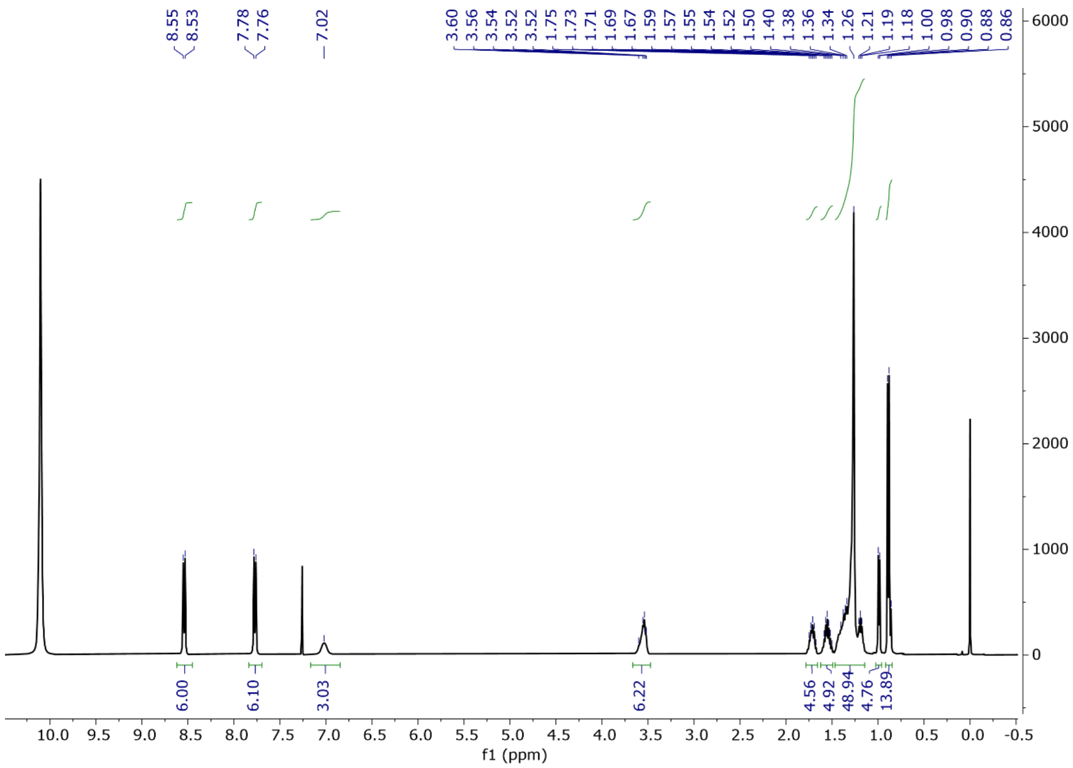

Figure S1. ^{1}H-NMR spectrum (400 MHz, Chloroform-d_1) of ***S*-T(R)**.

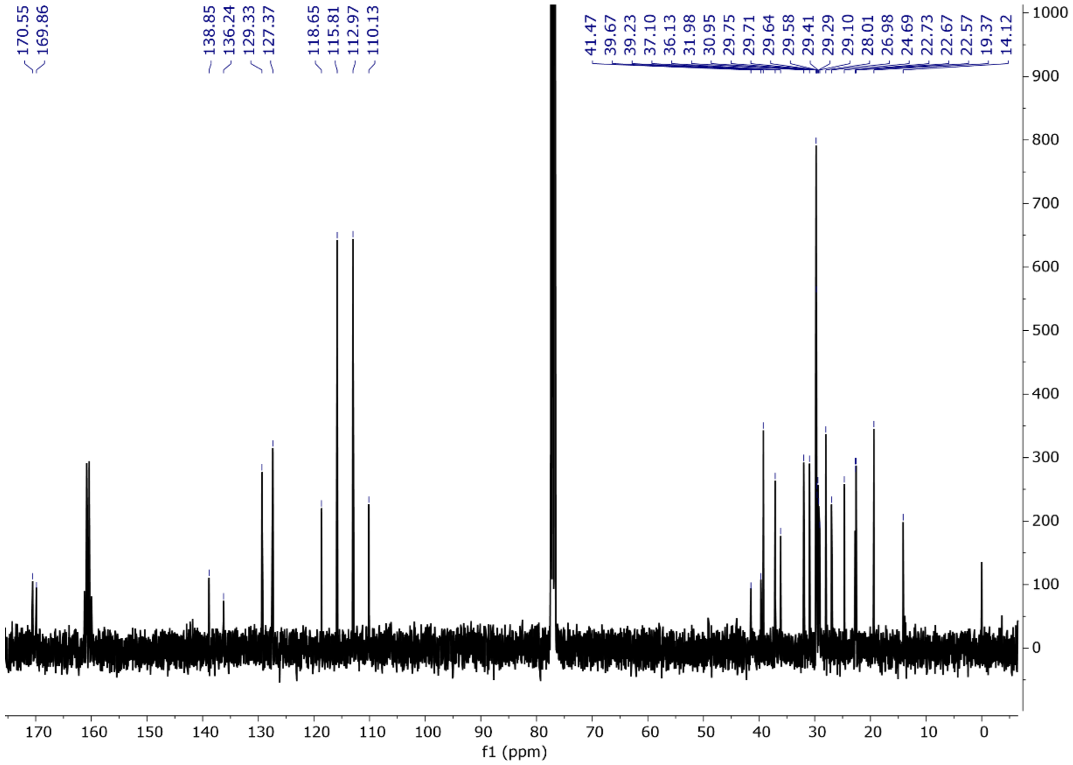

Figure S2. ^{13}C-NMR spectrum (101 MHz, Chloroform-d_1) of ***S*-T(R)**.

S2.2 Synthesis of N4,N4 ''-bis((S)-3,7-dimethyloctyl)-5'-(4-(((S)-3,7-dimethyloctyl)carbamoyl) phenyl)-[1,1':3',1''-terphenyl]-4,4''-dicarboxamide (*S*-B)

Chiral *S*-triphenyl benzene (***S*-B**) was synthesized following a previously reported procedure.[1]

^{1}H NMR (400 MHz, Chloroform-d_1): δ [ppm] = 7.88 (d, J = 8.3 Hz, 6H), 7.80 (s, 3H), 7.74 (d, J = 8.3 Hz, 6H), 6.14 (t, J = 5.6 Hz, 3H), 3.60 – 3.43 (m, 6H), 1.73 – 1.65 (m, 3H), 1.61 – 1.41 (m, 9H), 1.37 – 1.13 (m, 18H), 0.97 (d, J = 6.5 Hz, 9H), 0.87 (d, J = 6.6 Hz, 18H). ^{13}C NMR (101 MHz, Chloroform-d_1) δ [ppm]: 166.99, 143.54, 141.62, 134.10, 127.52, 127.44, 125.74, 39.24, 38.36, 37.17, 36.81, 30.85, 27.97, 24.69, 22.71, 22.61, 19.59.

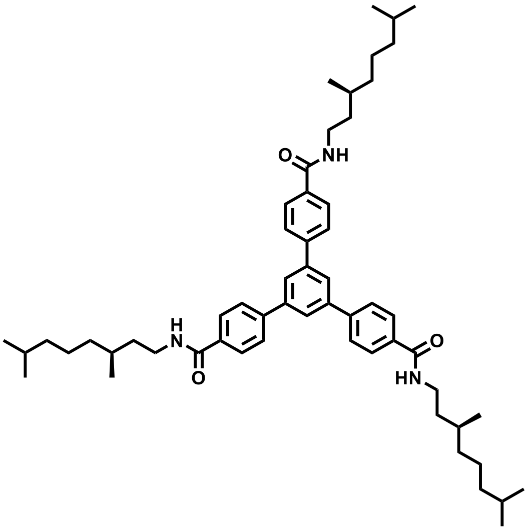

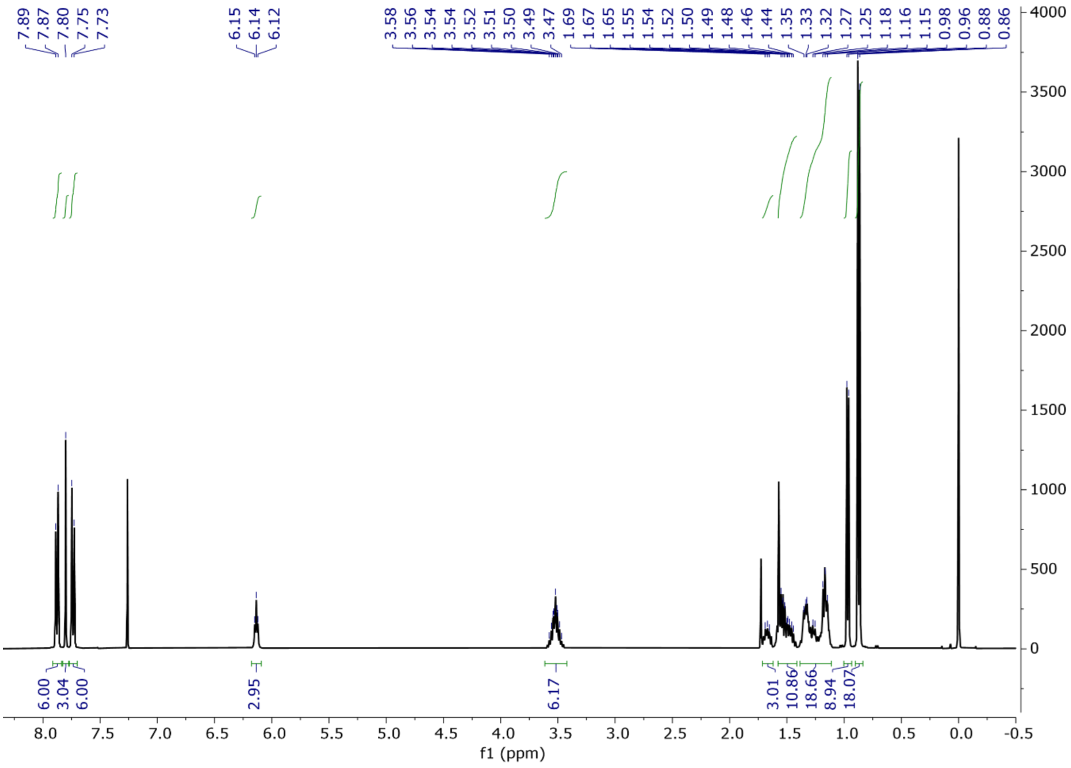

Figure S3. ^{1}H-NMR spectrum (400 MHz, Chloroform-d_1) of ***S*-B**.

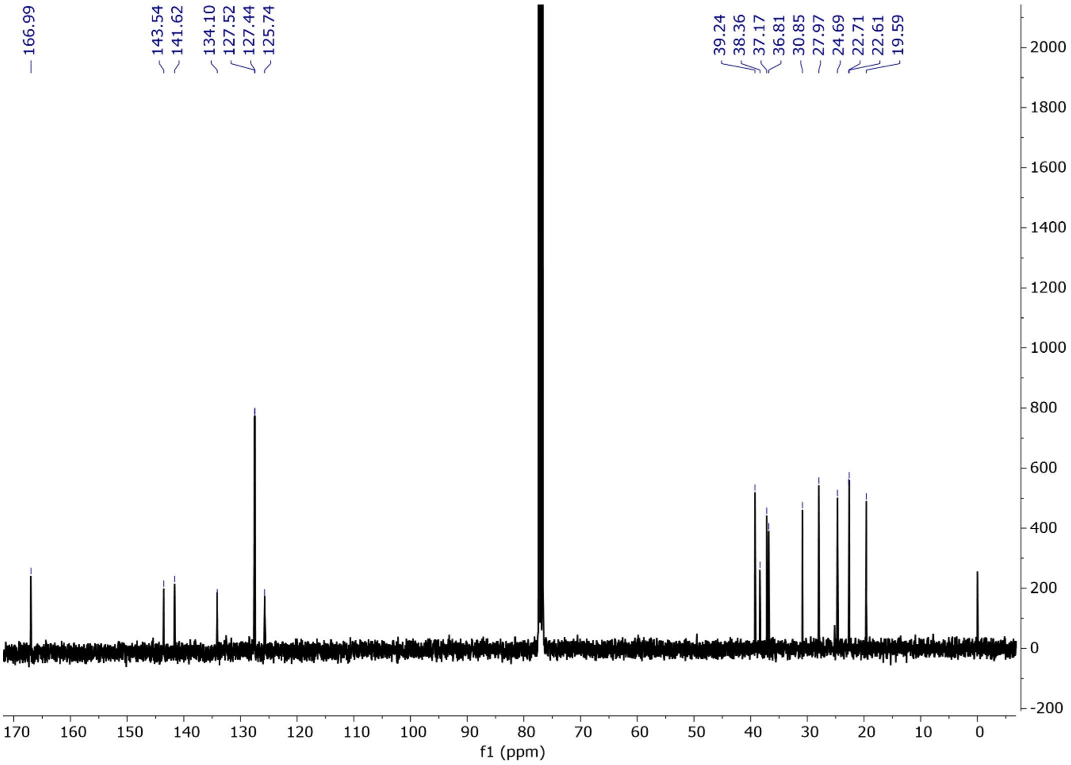

Figure S4. ^{13}C-NMR spectrum (101 MHz, Chloroform-d_1) of ***S*-B**.

3. Macromolecule-solvent system selection

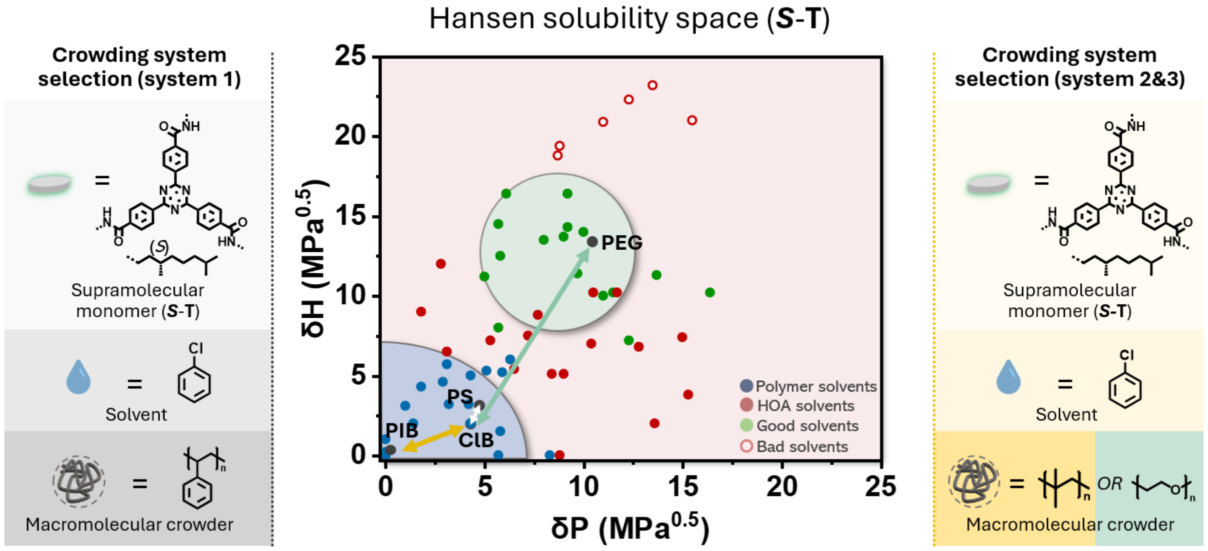

Figure S5. A 2D representation of the Hansen solubility space of ***S*-T** at 100 µM including respective HSPs of the predominantly used solvent-macromolecule system in the Hansen solubility space (white arrow). The HSPs of chlorobenzene (δ_D = 19.0, δ_P = 4.3, δ_H = 2.0), polystyrene (δ_D = 18.5, δ_P = 4.5, δ_H = 2.9), polyisobutylene (δ_D = 18.0, δ_P = 0.0, δ_H = 1.0) and polyethylene glycol (δ_D = 21.5, δ_P = 10.9, δ_H = 13.1).[6]

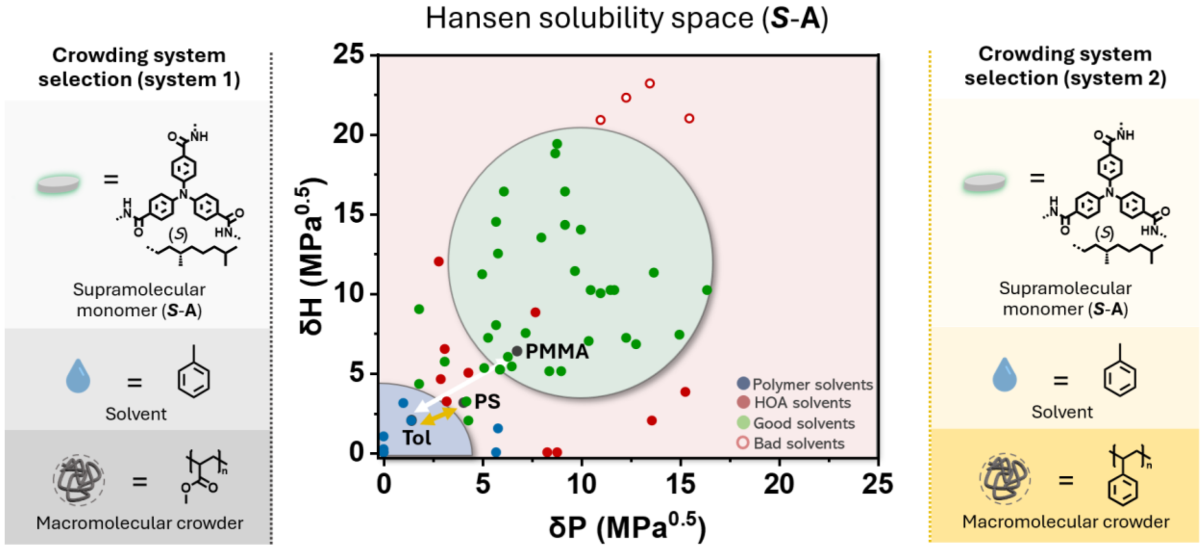

Figure S6. A 2D representation of the Hansen solubility space of ***S*-A** at 100 µM including respective HSPs of the solvent-macromolecule system 1 (white arrow) and system 2 (yellow arrow) in the Hansen solubility space. The HSPs of system 1 [toluene (δ_D = 18.0, δ_P = 1.4, δ_H = 2.0)] and polystyrene (δ_D = 18.5, δ_P = 4.5, δ_H = 2.9)] and system 2 [toluene (δ_D = 18.0, $p_{P.}$ = 1.4, δ_H = 2.0)] and polymethyl methacrylate (δ_D = 17.7, δ_P = 6.7, δ_H = 6.5)].[6]

4. Thermodynamic versus kinetically controlled CD measurements

S4.1 Sample preparation & measurement procedure

Sample preparation. Stock solutions of PS (20 wt%) and ***S*-T** (1 mM or 200 µM) were prepared in dried chlorobenzene (filtered over basic Al_2O_3). Subsequently, the desired amount of PS stock solution was transferred into a separate 3 mL vial followed by adjusting the total volume to 0.95 mL with additional chlorobenzene. To acquire a final ***S*-T** concentration of 50 or 10 µM, 50 µL of the corresponding 1 mM or 200 µM stock solution of ***S*-T** was added. This resulted in a constant total volume and varying concentrations of PS crowder (1 to 20 wt%). Following the addition of each component, the sample was heated to 80 °C and vortexed to ensure thorough mixing. The same procedure was performed prior to transferring the sample into a 1 mm sealed cuvette. To minimize water contamination, all CD and UV measurements were performed using freshly prepared solutions, within a maximum of one month after preparation.

Thermodynamically controlled procedure. For the thermodynamically controlled measurements, the samples were initially heated to 90 °C and equilibrated for 5 minutes. The procedure is followed by a slow cooling process at a rate of 1 °C min^{-1} until reaching room temperature, prior to recording a CD spectrum.

Kinetically controlled procedure. For the kinetically controlled measurements, the solutions were first heated to 90 °C and equilibrated for 5 minutes. Opposed to the thermodynamically controlled procedure, the samples were cooled with a rate of 10 °C min^{-1} to room temperature. However, as the 10 °C min^{-1} is practically not feasible on the JASCO J-815 CD spectrometer, CD spectra are recorded exactly 10 minutes after starting the cooling ramp (t = 0). Subsequently, the CD spectra are acquired at fixed time points.

S4.2 Cooperative supramolecular polymerization of *S*-T in uncrowded media.

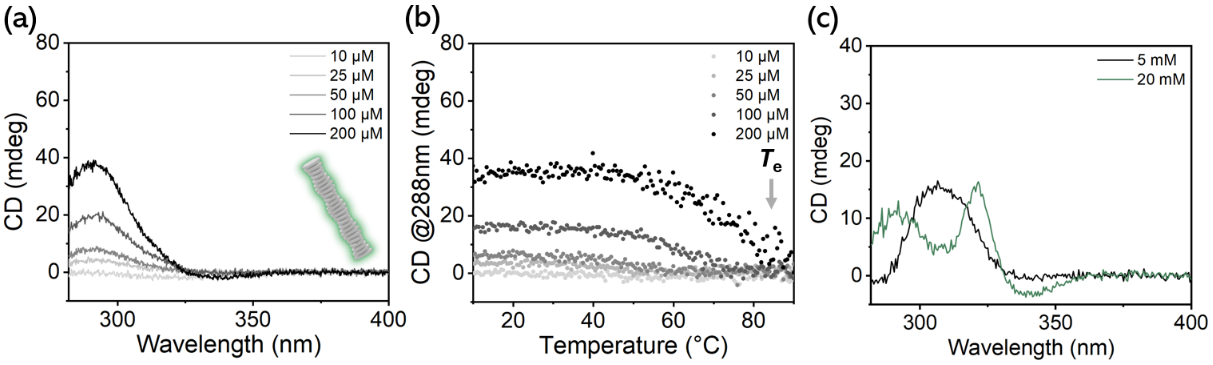

Figure S7. (a) CD spectra and (b) the corresponding cooling curves of ***S*-T** at various concentrations. (c) CD spectra at comparatively high total concentrations of ***S*-T**. All spectra were recorded in uncrowded media with chlorobenzene as solvent. All cooling runs were performed at 1 °C min^{-1} and recorded at 288 nm. The T_e represents the elongation temperature for supramolecular polymerization. Samples presented in (a) and (b) were measured with a 1 mm cuvette, while samples in (c) were measured with a 0.1 mm cuvette to prevent detector oversaturation.

S4.3 Thermodynamically versus kinetically controlled CD measurements of crowded solutions of *S*-T

As discussed in the results and discussion section of the main text, solutions with 5 wt% of crowder and below did not display any CD signal (Figure 1d). Yet, solutions with 10 wt% of crowder and above showcased a pronounced Cotton effect, indicative of supramolecular polymerization and consequent

bundling into higher-order aggregates (HOAs) of ***S*-T**. The fact that the supramolecular polymerization merely occurs at high crowder concentrations (≥ 10 wt%) is indicative of an assembly process triggered by crowding (volume exclusion). The sequential order of assembly events into HOAs is clear from the ***S*-T** cooling curve in the presence of 10 wt% of crowder, which displays both an elongation temperature (T_e) and a critical temperature (T_c) at which HOAs form (Figure S8a-b). These two clear onsets upon cooling may be indicative of a secondary nucleation event. However, this study does not conclusively demonstrate whether the preformed supramolecular polymers function as secondary nucleation sites for monomer growth or whether hierarchical assembly leads to the bundling of already formed supramolecular assemblies. Figure S8b depicts the latter mechanism.

Interestingly, ***S*-T** solutions containing 15 and 20 wt% of crowder only exhibit a T_c, as the T_c exceeds the T_e in these conditions. Moreover, the T_c of crowded 50 µM ***S*-T** solutions increases with increasing content of crowder, which is attributed to the increasing effective concentration of ***S*-T** as the crowder continues to exclude more volume.

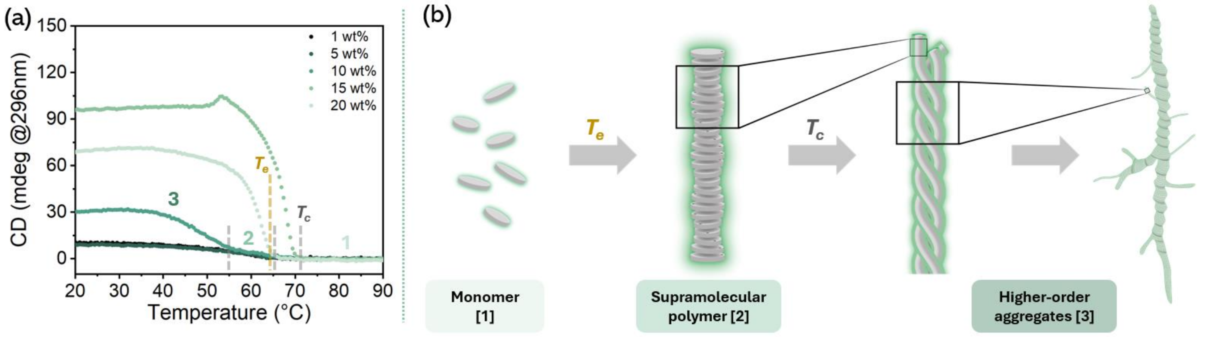

Figure S8. (a) CD spectra and (b) respective cooling curves of crowded 10 µM ***S*-T** solutions at various concentrations of PS (MW = 50 kDa) in chlorobenzene. All cooling runs were performed at 1 °C min^{-1} and recorded at 296 nm. The T_c represents the critical assembly temperature at which higher-order aggregates form.

The formation of HOAs, however, is highly influenced by the cooling rate of the solution, as can be derived from the varying shapes and intensities of the cooling curves and respective CD spectra with increasing crowder content (Figure S9). We attribute these variable CD spectra to different types of HOAs being formed, demonstrating the challenging task to control the assembly processes of ***S*-T** in crowded environments. As depicted in Figure S9c,f, the highest cooling rate (10 ºC min^{-1}) provided the most reproducible CD spectra, opposed to conventional supramolecular polymerizations in apolar solvents for which the probability of forming kinetic traps rises with increasing cooling rate. We therefore continued to apply a cooling rate of 10 ºC min^{-1} for all other measurements.

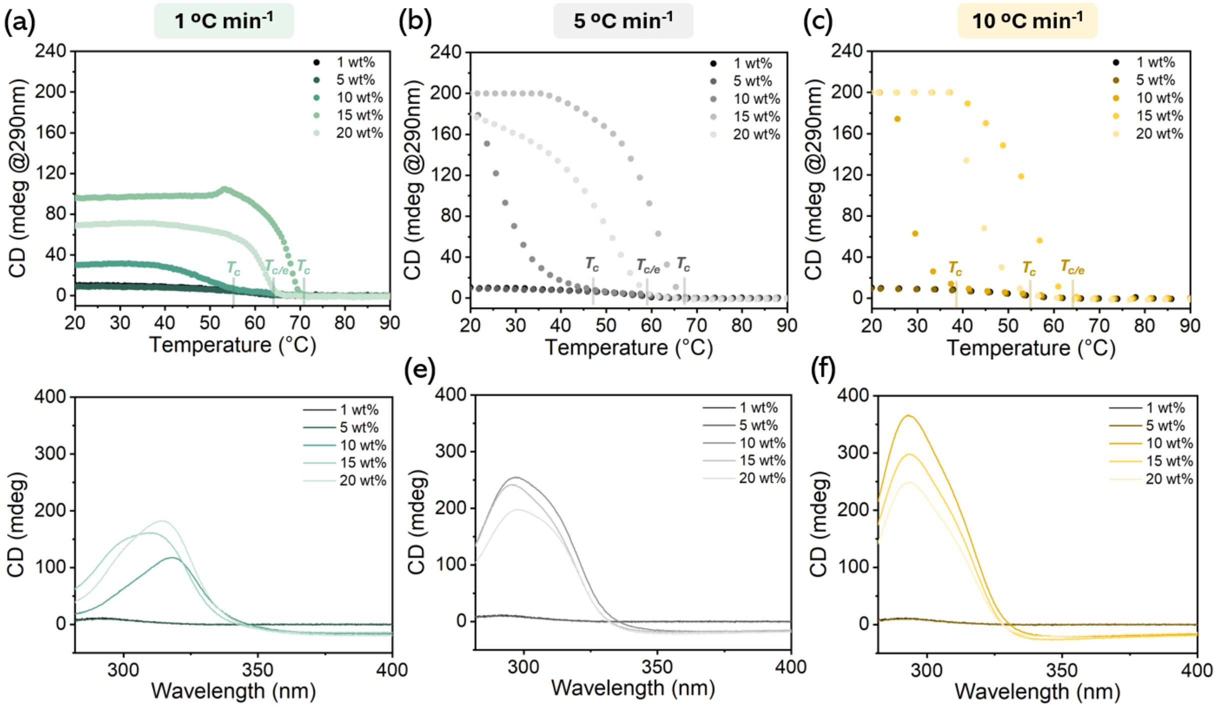

Figure S9. (a–c) Cooling curves and (d–f) respective CD spectra of crowded 10 µM ***S*-T** solutions at various concentrations of PS (MW = 50 kDa) in chlorobenzene for cooling rates of (a+d) 1 ºC min^{-1}, (b+e) 5 ºC min^{-1} and (c+f) 10 ºC min^{-1}. The CD signal in all cooling runs were recorded at 290 nm. The T_e represents the elongation temperature for supramolecular polymerization, while the T_c indicates the critical temperature at which higher-order aggregates are formed.

Upon reaching room temperature, after cooling with 10 ºC min^{-1} (Figure S10b), the supramolecular polymerization and/or bundling into HOAs of ***S*-T** continues to proceed over a time (Figure S10c). After roughly 60 minutes, the consistent CD spectra imply that a relatively stable thermodynamic state appears to have been reached (Figure S10d). Motivated by these results, we decided to compare the CD spectra in crowded solutions merely after 0 and/or 60 minutes in order to maintain reproducibility, consistency and create a fair comparison between different conditions. An example of such a comparison is illustrated in Figure S10e and is compiled by extracting the maximum CD signal from the obtained spectra at t = 60 minutes followed by plotting those values versus the crowder concentration.

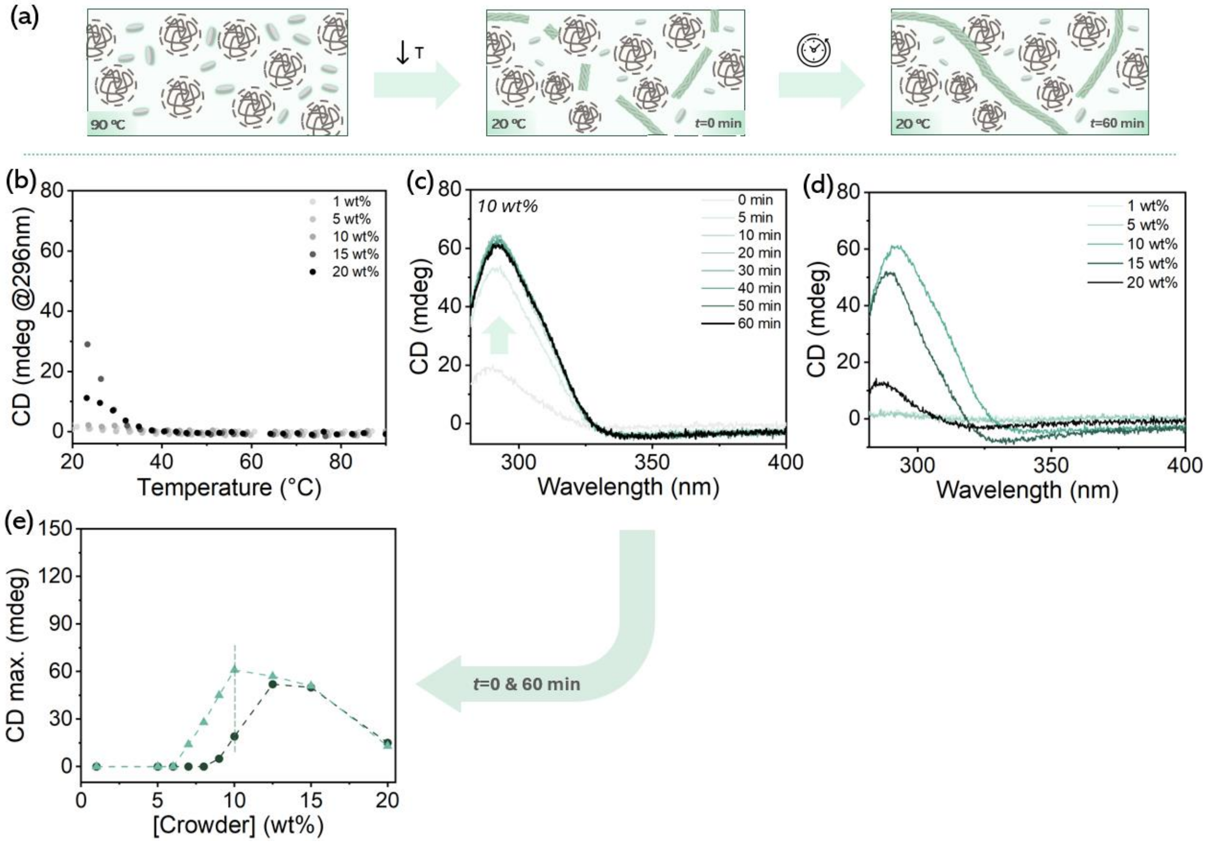

Figure S10. (a) Schematic representation of the kinetically controlled supramolecular polymerization in a crowded environment. (b) Cooling curves (followed at 296 nm) of 10 μM ***S*-T** solutions at various concentrations of PS crowder in chlorobenzene. A cooling rate of 10 °C min^{-1} was used. (c) CD spectra of a 10 μM ***S*-T** solution containing 10 wt% PS in chlorobenzene at fixed time points. (d) CD spectra of a 10 μM ***S*-T** solution at various concentrations of PS crowder in chlorobenzene after 60 minutes. (e) The maximum observed CD signal, extracted from plot (c), as function of the crowder concentration for a 10 μM solution of ***S*-T**.

5. CD data of supramolecular polymerizations in crowded media

S5.1 CD versus crowder concentration for crowded *S*-T media.

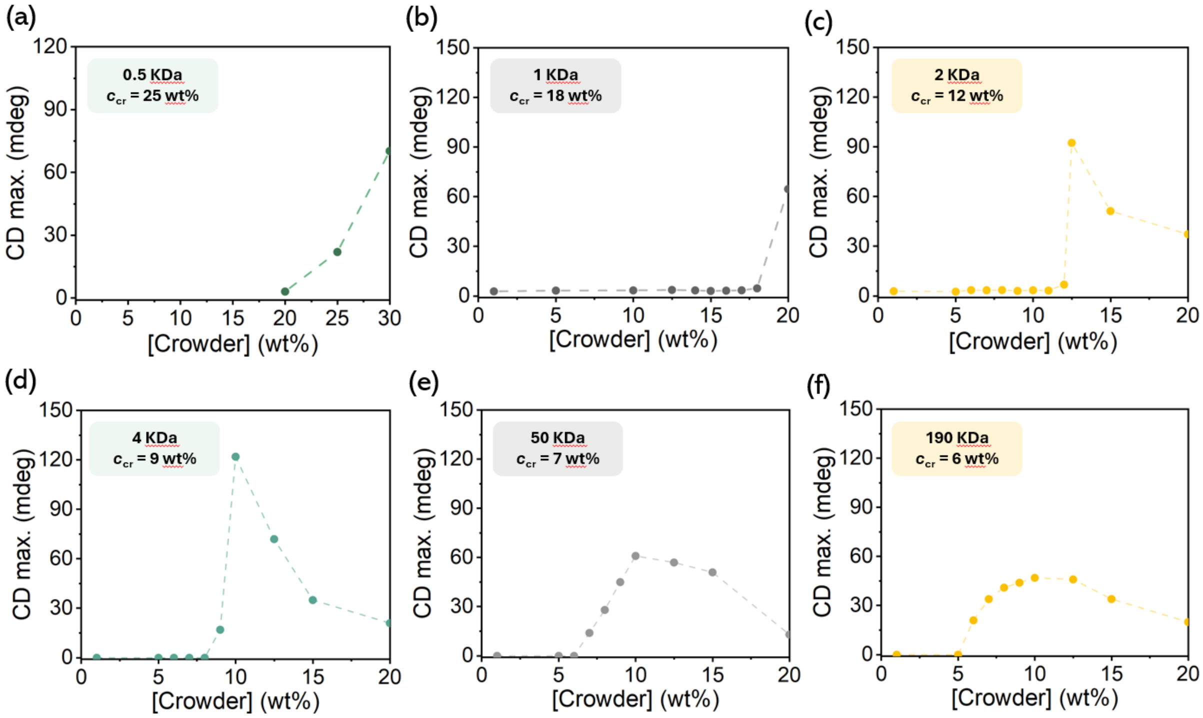

Figure S11. The maximum observed CD signal as function of the crowder concentration for 10 µM solutions of ***S*-T** containing PS with a Mw of (a) 0.5 kDa, (b) 1 kDa (c) 2 kDa (d) 4 kDa, (e) 50 kDa and (f) 190 kDa. All experiments are performed in chlorobenzene using a kinetically controlled approach and data points are extracted from t = 60 minutes.

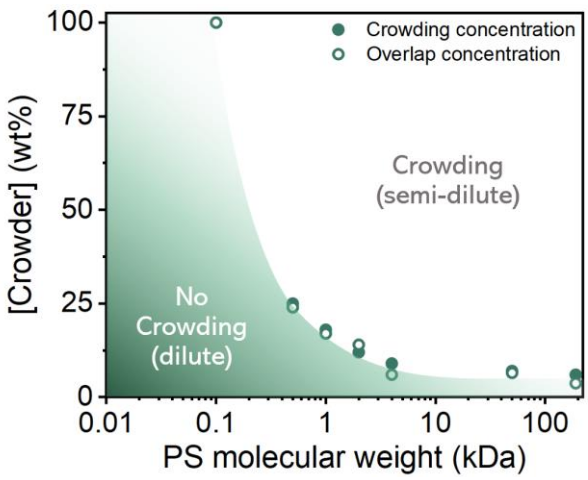

Figure S12. A comparison between the overlap concentration (c*) of polystyrene in chlorobenzene and the crowding concentration (c_{cr}) of 10 µM solutions of ***S*-T** in chlorobenzene with polystyrene as a crowder.

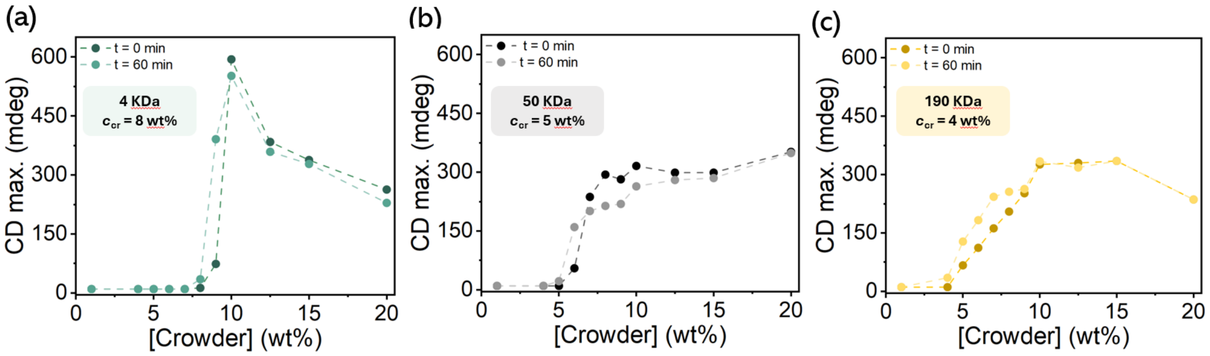

Figure S13. The maximum observed CD signal as function of the crowder concentration for 50 µM solutions of ***S*-T** containing PS with a Mw of (a) 4 kDa, (b) 50 kDa and (c) 190 kDa. All experiments are performed in chlorobenzene using a kinetically controlled approach.

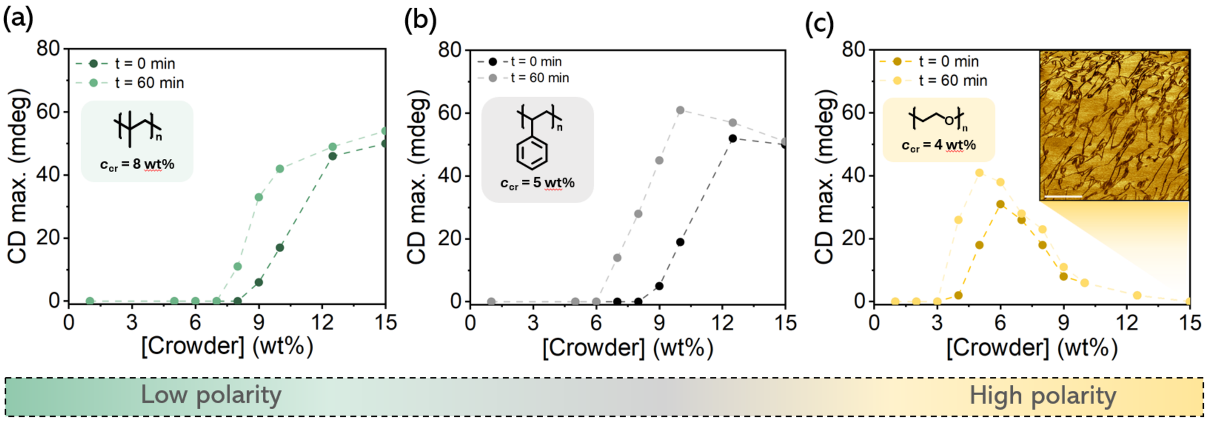

Figure S14. The maximum observed CD signal as function of the crowder concentration for 10 µM solutions of ***S*-T** containing (a) polyisobutylene (b) polystyrene and (c) polyethylene glycol as crowders. All experiments are performed in chlorobenzene using a kinetically controlled approach. The inset figure in (c) represents a 20x20 µm AFM phase image of spincoated 10 µM solution containing 15 wt% PEG crowder taken at 70 °C. The image clearly displays the presence of supramolecular polymers, indicating that the disappearance of CD signal is not caused by depolymerization of ***S*-T**.

S5.2 The macromolecular crowding effect for various supramolecular building blocks.

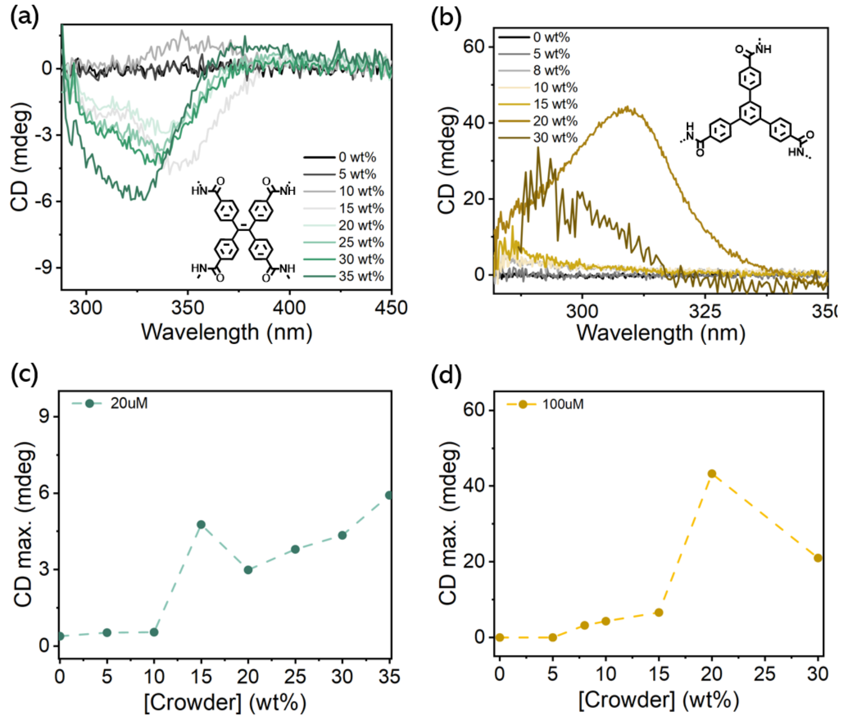

Figure S15. CD spectra for crowded solutions of (a) 20 µM ***S*-E** in chlorobenzene and (b) 100 µM ***S*-B** in toluene at various concentrations of PS crowder after 60 minutes. (c) and (d) represent respective maximum CD signals as function of the crowder concentration. All experiments are performed with polystyrene (MW of 50 kDa) as the crowder.

6. The correlation between crowder regimes and evolution of CD

S6.1 Specific viscosity data of various crowder systems.

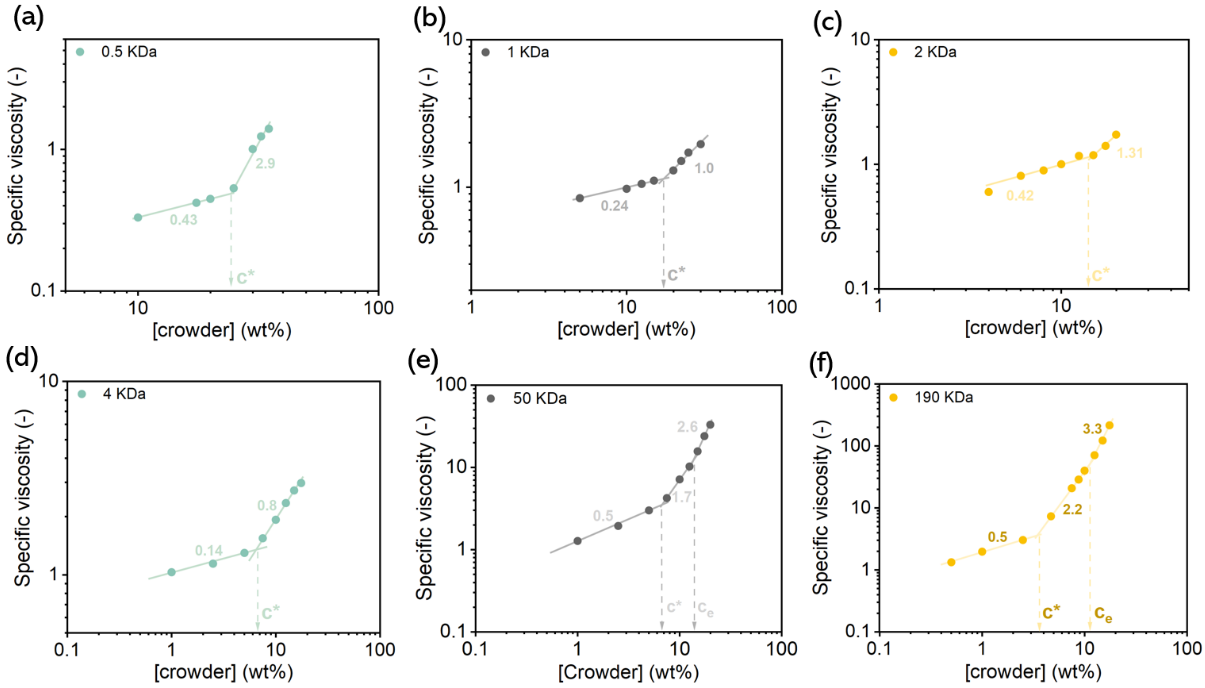

Figure S16. Specific viscosity plotted against the concentration of polystyrene with a molecular weight of (a) 0.5 kDa, (b) 1 kDa (c) 2 kDa (d) 4 kDa, (e) 50 kDa and (f) 190 kDa. The overlap and entanglement concentrations for each Mw of polystyrene are determined by the inflection points in the viscosity curve, as indicated by the arrows.

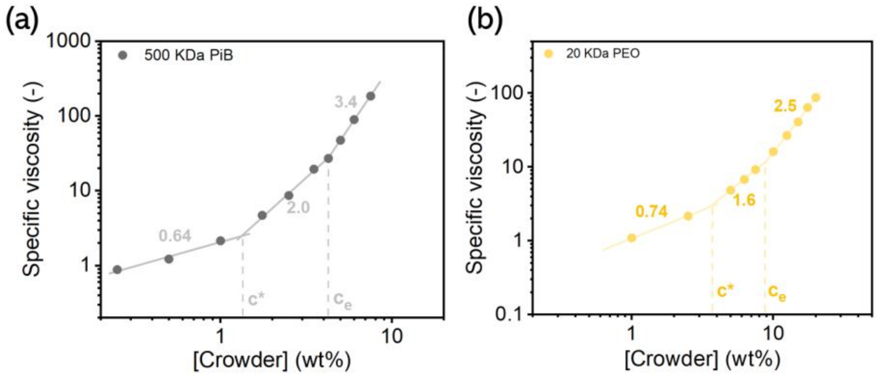

Figure S17. Specific viscosity plotted against the concentration of (a) polyisobutylene and (b) polyethylene glycol in chlorobenzene. The overlap and entanglement concentrations for each crowder are determined by the inflection points in the viscosity curve, as indicated by the dotted lines.

Table S2. Overview of the crowding-, overlap- and entanglement concentrations of the employed crowding systems.

Crowder [a]	Solvent [b]	c_{cr} [c]	c* [wt%] [d]	c_e [wt%] [e]
**PS(0.5)**	Chlorobenzene	25	24	-[f]
**PS(1)**	Chlorobenzene	18	17	-[f]
**PS(2)**	Chlorobenzene	12	14	-[f]
**PS(4)**	Chlorobenzene	9	6.7	-[f]
**PS(50)**	Chlorobenzene	7	6.6	14
**PS(190)**	Chlorobenzene	6	3.7	11.3
**PEG (20)**	Chlorobenzene	4	3.7	8.7
**PIB (500)**	Chlorobenzene	8	1.3	4.3

[a] Polymers, as denoted in table S1, and [b] solvents describing the polymer solutions used for the viscosity measurements. [c] Crowding concentration (c_{cr}) of ***S*-T** in the respective crowding systems. [d] Overlap concentration (c*) and [e] entanglement concentration (c_e) of the studied polymer solutions. [f] Polymer solutions without an experimentally observed c_e.

S6.2 The evolution of CD over time for various crowder concentrations.

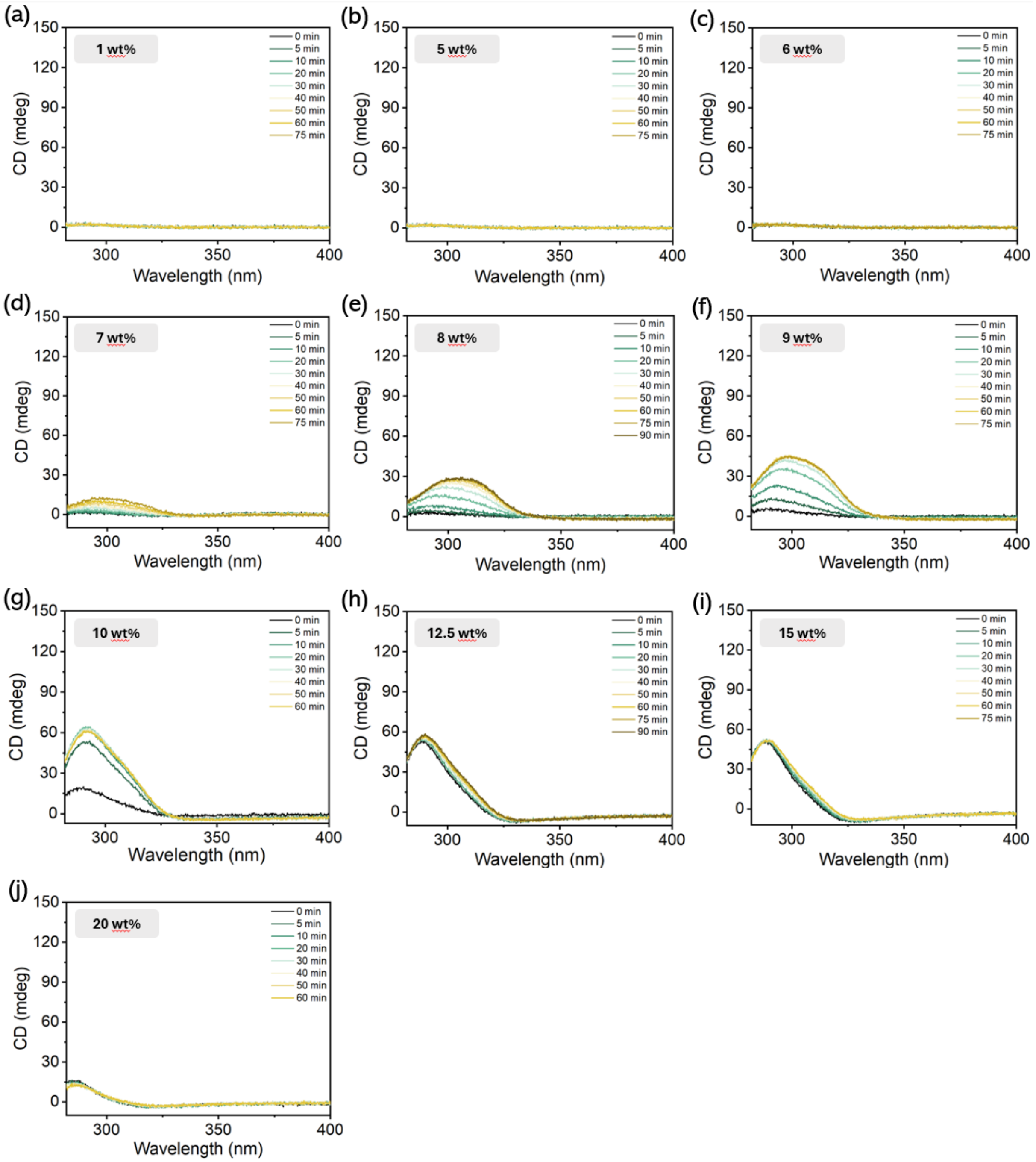

Figure S18. CD as function of the crowder concentration recorded at various points in time for (a) 1 wt%, (b) 5 wt%, (c) 6 wt%, (d) 7 wt%, (e) 8 wt%, (f) 9 wt%, (g) 10 wt%, (h) 12.5 wt%, (i) 15 wt% and (j) 20 wt% of crowder. All experiments were performed with 10 µM ***S*-T** solutions using polystyrene (50 kDa) as the crowder.

In Figure S18, the CD is presented as a function of the crowder concentration, recorded at various time points. At relatively low concentrations of crowder (<6 wt%), no crowding effect is observed. However, at crowder concentrations exceeding 6 wt%, the CD increases over time, indicative of crowding-induced supramolecular polymerization and bundling. This time-dependent increase in the CD signal is absent for c_{cr} exceeding 12.5 wt%, due to the increasingly rapid kinetics of the sequential polymerization and bundling of ***S*-T** into HOAs. This is evident in Figures S9c and S10b, where the increase in the CD signal becomes progressively faster at higher crowder concentrations. Hence, at concentrations greater

than 12.5 wt%, the CD signal has already reached its maximum by the time the kinetic measurements start.

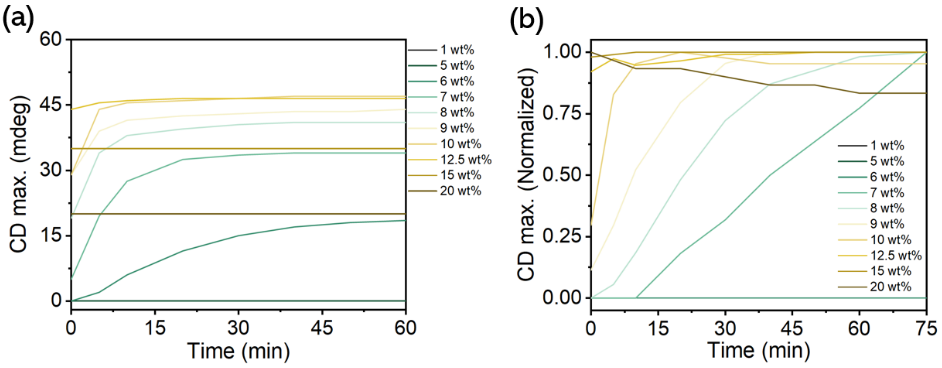

Figure S19. (a) The evolution of CD as function of time for various crowder concentrations and (b) respective normalized spectra. All experiments were performed with 10 µM ***S*-T** solutions using polystyrene (50 kDa) as the crowder.

7. Morphology of crowded solutions of *S*-T & *S*-E

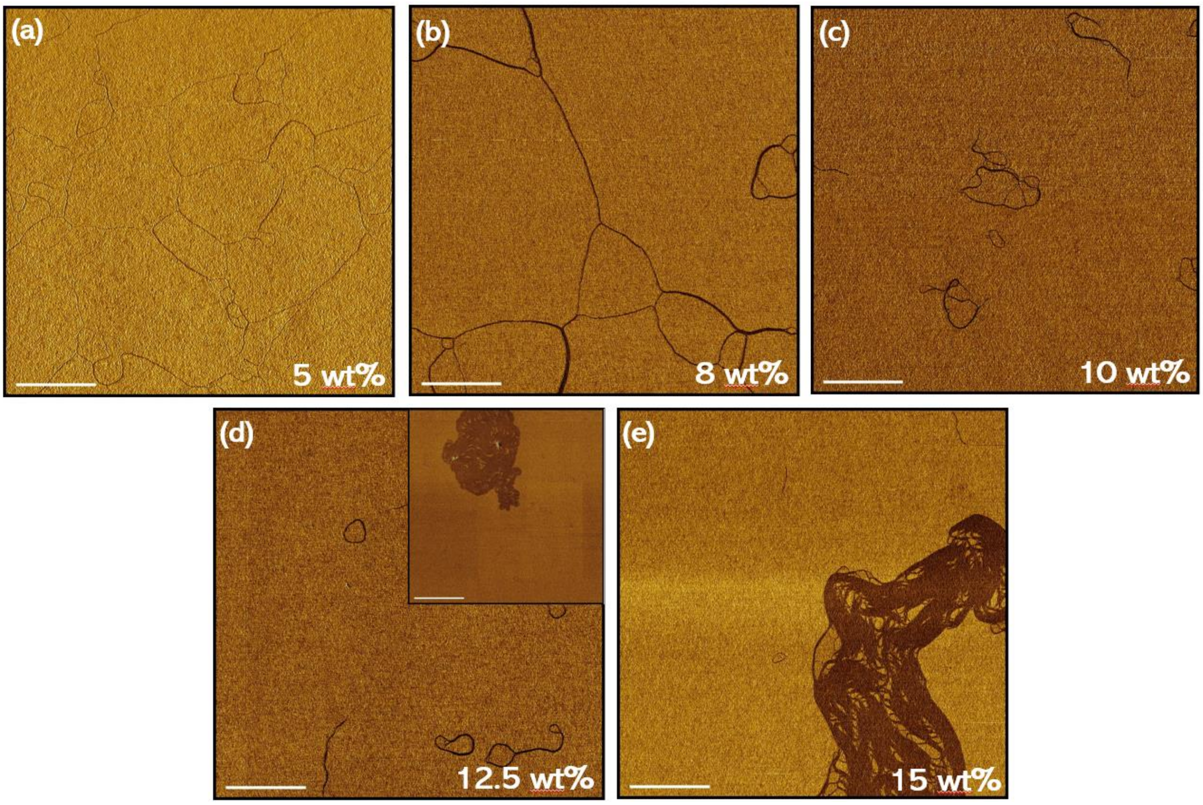

Figure S20. 10x10 µm AFM phase images of spincoated samples of 10 µM solutions of ***S*-T** in chlorobenzene with (a) 5 wt%, (b) 8 wt%, (c) 10 wt%, (d) 12.5 wt% and (e) 15 wt% of PS crowder. All inset scalebars represent 2 µm.

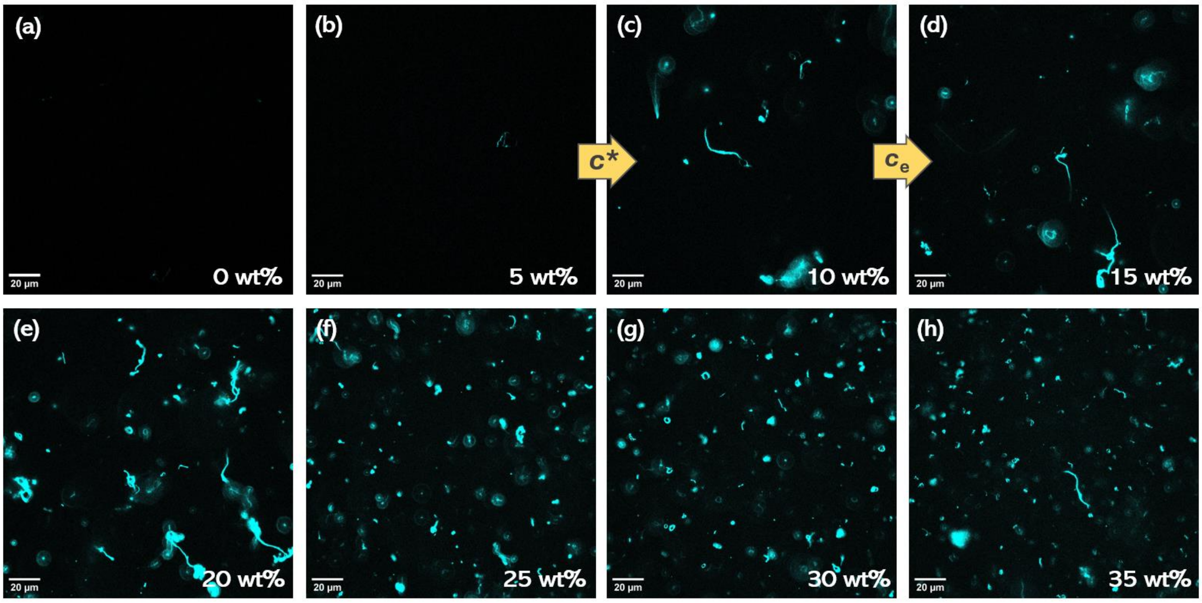

Figure S21. 10x10 µm AFM phase images of spincoated samples of 10 µM solutions of ***S*-T** in chlorobenzene with (A) 5 wt%, (B) 8 wt%, (C) 10 wt%, (D) 12.5 wt% and € 15 wt% of PS crowder. All inset scalebars represent 2 µm.

8. CD and infrared spectroscopy of highly concentrated solutions of *S*-B.

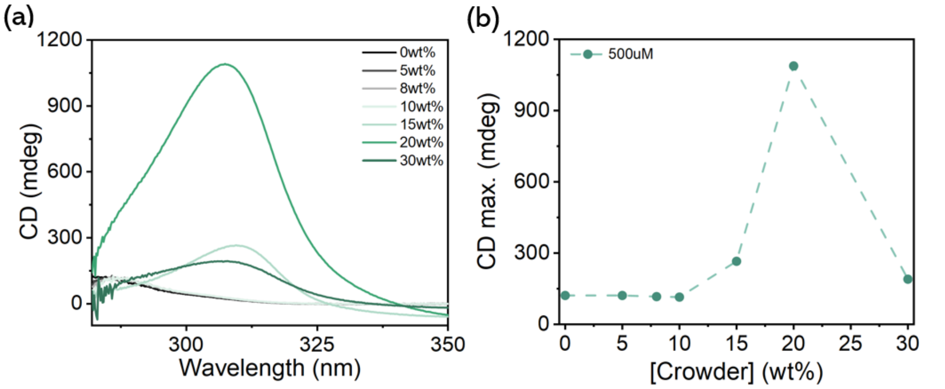

Figure S22. (a) CD spectra of crowded solutions of 500 µM ***S*-B** in toluene at various concentrations of PS crowder after 60 minutes. (b) Maximum observed CD signal as function of the crowder concentration. All experiments are performed with polystyrene (MW of 50 kDa) as the crowder.

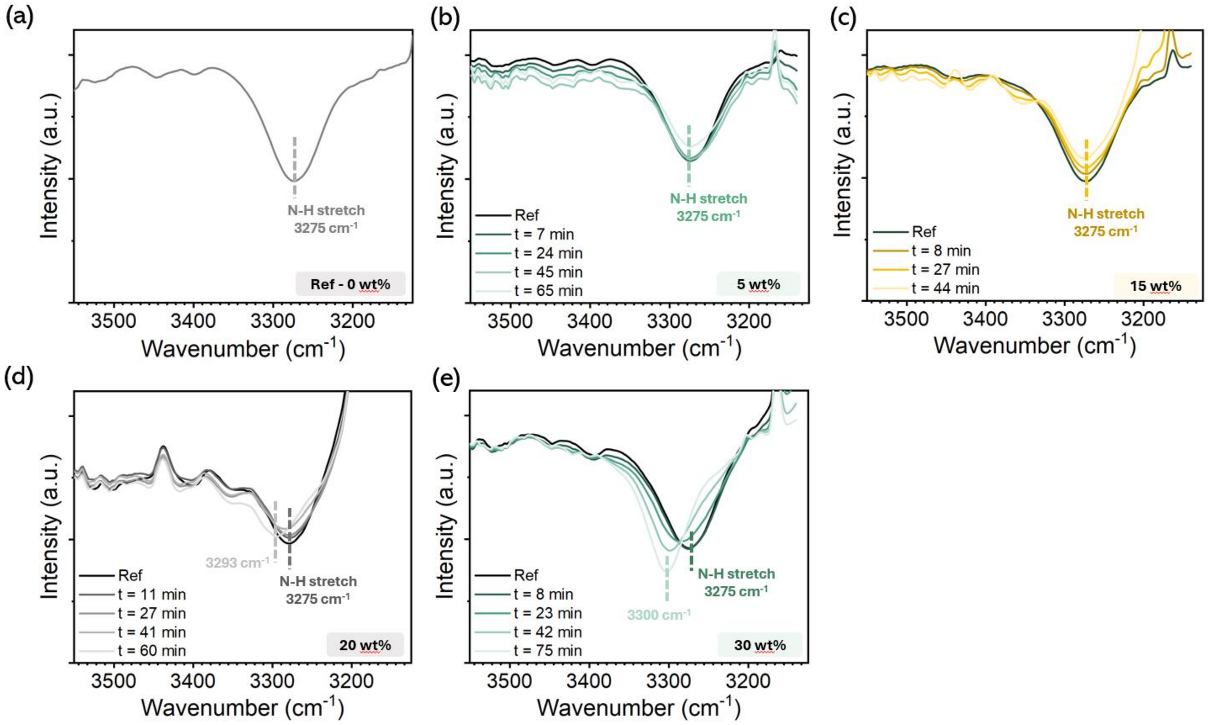

Figure S23. Fourier-transform infrared spectra of crowded solutions of ***S*-B** followed over time for crowder concentrations of (a) 0 wt%, (b) 5 wt%, (c) 15 wt%, (d) 20 wt% and (e) 30 wt%. All spectra were recorded on a 500 µM solution of ***S*-B** using polystyrene (50 kDa) as crowder and following the kinetically controlled sample preparation as described in section S4. All spectra are shifted along the y-axis for alignment and clarity. The carbonyl stretch region of the FTIR spectra is not shown due to a large overlap with the C=C stretch of PS crowder, resulting in unreliable results.

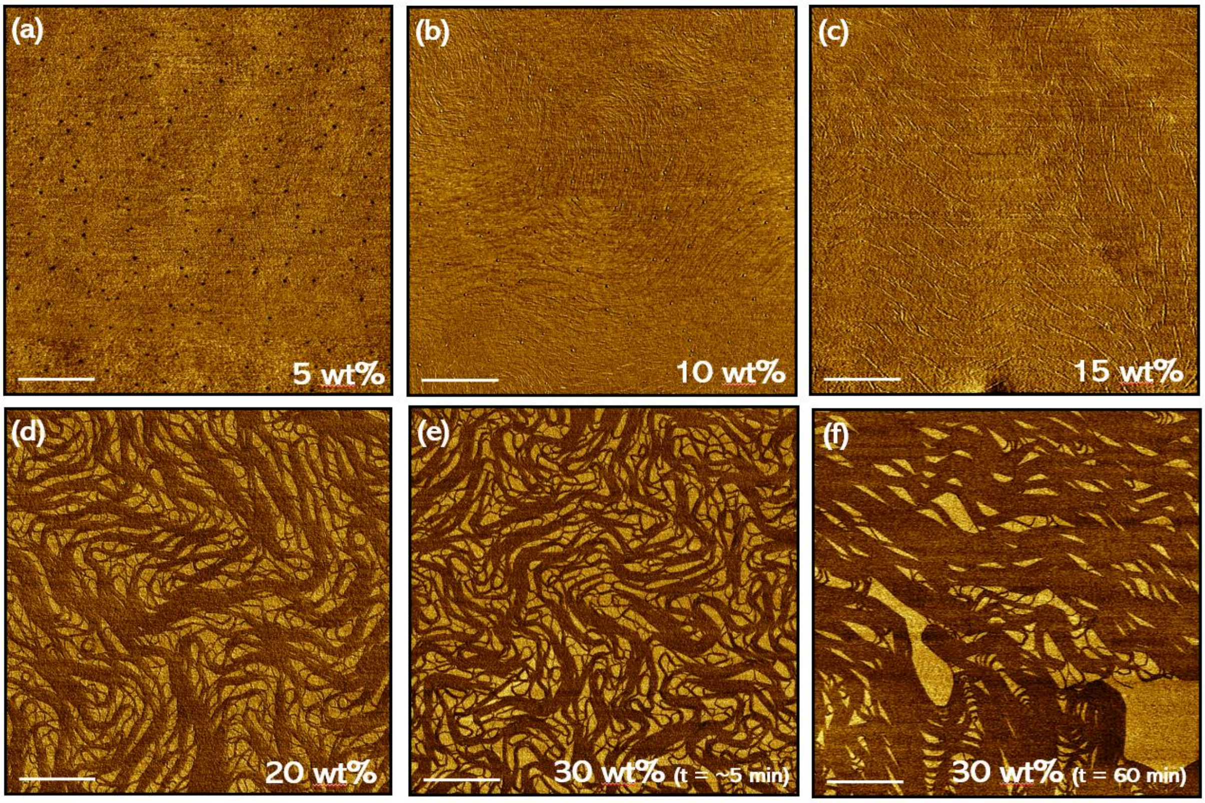

Figure S24. 10x10 µm AFM phase images of spincoated samples of 500 µM solutions of ***S*-B** in toluene with PS crowder concentrations of (A) 5 wt%, (B) 10 wt%, (C) 15 wt%, (D) 20 wt%, (E) 30 wt% at *t* = ~5 min, and (F) 30 wt% at *t* = 60 min. All inset scalebars represent 2 µm.

9. Spectroscopic characterization of *S*-T(R) in uncrowded and crowded media

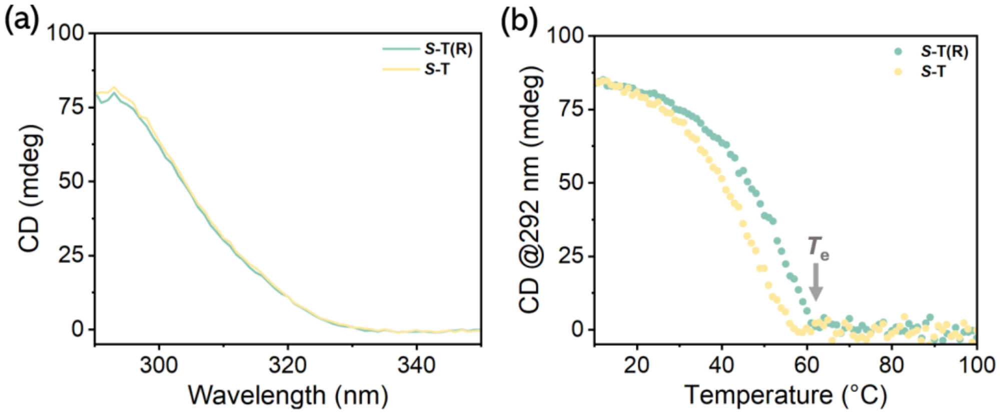

Figure S25. (a) CD spectra and (b) corresponding cooling curves of 40 µM ***S*-T** and ***S*-T(R)** in uncrowded media with chlorobenzene as solvent. All cooling runs were performed at 1 °C min^{-1} and recorded at 292 nm. The T_e represents the elongation temperature for supramolecular polymerization.

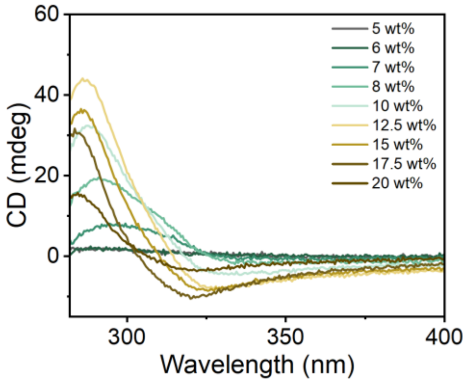

Figure S26. CD spectra of crowded 10 µM solutions of ***S*-T(R)** in chlorobenzene at various concentrations of PS crowder after 60 minutes. In all experiments PS with a MW of 190 kDa is used as crowder.

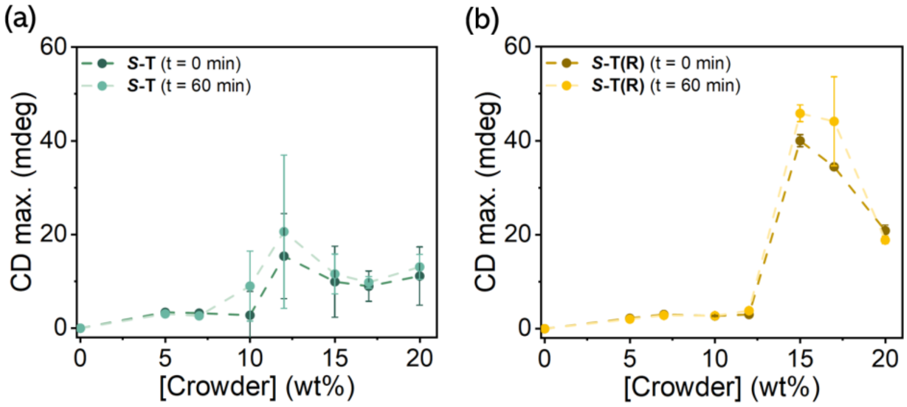

Figure S27. The maximum observed CD signal as function of the crowder concentration for crowded 10 µM (a) ***S*-T** and (b) ***S*-T(R)** solutions in chlorobenzene. In all experiments PS with a MW of 50 kDa is used as crowder.

10. Sequestration versus crowding of supramolecular building block *S*-A

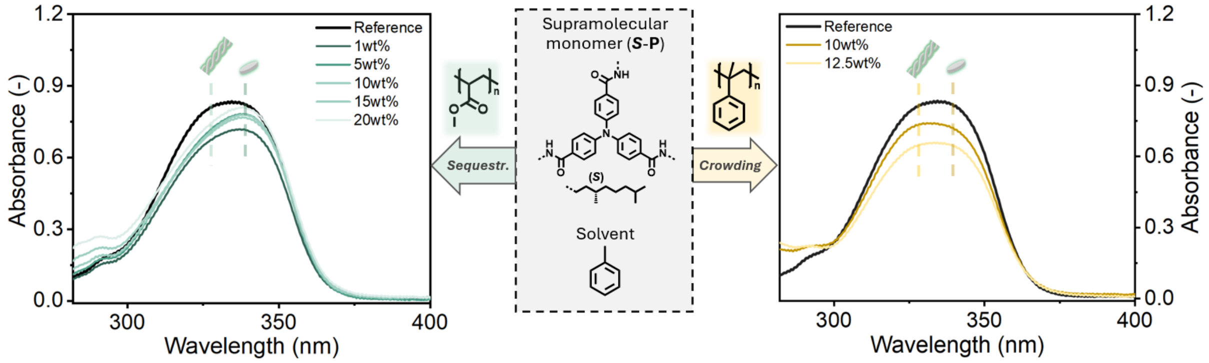

Figure S28. UV spectra of ***S*-A** (250 µM) in the presence of a sequestrator (PMMA, left) and crowder (PS, right). In both cases, toluene is used as a solvent to promote supramolecular polymerization. All spectra are shifted vertically to correct for elevated baselines as a consequence of scattering at high concentrations of PS crowder.

11. References

(1) Su, H.; Jansen, S. A. H.; Schnitzer, T.; Weyandt, E.; Andreas, T. R.; Liu, J.; Vantomme, G.; Meijer, E. W. Unraveling the Complexity of Supramolecular Copolymerization Dictated by Triazine − Benzene Interactions. *J. Am. Chem. Soc.* **2021**, *143*, 17128–17135. https://doi.org/10.1021/jacs.1c07690.

(2) Adelizzi, B.; Filot, I. A. W.; Palmans, A. R. A.; Meijer, E. W. Unravelling the Pathway Complexity in Conformationally Flexible N-Centered Triarylamine Trisamides. *Chem. - A Eur. J.* **2017**, *23* (25), 6103–6110. https://doi.org/10.1002/chem.201603938.

(3) Adelizzi, B.; Aloi, A.; Van Zee, N. J.; Palmans, A. R. A.; Meijer, E. W.; Voets, I. K. Painting Supramolecular Polymers in Organic Solvents by Super-Resolution Microscopy. *ACS Nano* **2018**, *12* (5), 4431–4439. https://doi.org/10.1021/acsnano.8b00396.

(4) Lavarda, G.; Berghuis, A. M.; Joseph, K.; van der Tol, J. J. B.; Murai, S.; Gómez Rivas, J.; Meijer, E. W. Tunable Emission from H-Type Supramolecular Polymers in Optical Nanocavities. *Chem. Commun.* **2024**, *60* (20), 2812–2815. https://doi.org/10.1039/d3cc05877h.

(5) Cantekin, S.; de Greef, T. F. A.; Palmans, A. R. A. Benzene-1,3,5-Tricarboxamide: A Versatile Ordering Moiety for Supramolecular Chemistry. *Chem. Soc. Rev.* **2012**, *41* (18), 6125–6137. https://doi.org/10.1039/c2cs35156k.

(6) van der Tol, J. J. B.; Vantomme, G.; Meijer, E. W. Solvent-Induced Pathway Complexity of Supramolecular Polymerization Unveiled Using the Hansen Solubility Parameters. *J. Am. Chem. Soc.* **2023**, *145* (32), 17987–17994. https://doi.org/10.1021/jacs.3c05547.

Appendix A

Table S3. Tabulated overlap concentration (c^*) and crowding concentration (c_{cr}) data of all employed crowding systems in this study.

Crowder	Solvent	Supramolecular monomer	c* [wt%]	c_{cr} [wt%]
**PS(0.5)**	Chlorobenzene	***S*-T [10 µM]**	24	25
**PS(1)**	Chlorobenzene	***S*-T [10 µM]**	17	18
**PS(2)**	Chlorobenzene	***S*-T [10 µM]**	14	12
**PS(4)**	Chlorobenzene	***S*-T [10 µM]**	6.7	9
**PS(50)**	Chlorobenzene	***S*-T [10 µM]**	6.6	7
**PS(190)**	Chlorobenzene	***S*-T [10 µM]**	3.7	6
**PS(4)**	Chlorobenzene	***S*-T [50 µM]**	6.7	8
**PS(50)**	Chlorobenzene	***S*-T [50 µM]**	6.6	5
**PS(190)**	Chlorobenzene	***S*-T [50 µM]**	3.7	4
**PEG (20)**	Chlorobenzene	***S*-T [10 µM]**	3.7	4
**PIB (500)**	Chlorobenzene	***S*-T [10 µM]**	1.3	8
**PS(50)**	Chlorobenzene	***S*-E [20 µM]**	6.6	10-15
**PS(50)**	Toluene	***S*-B [100 µM]**	6.6	8-10
**PS(50)**	Toluene	***S*-B [500 µM]**	6.6	10-15
**PS(50)**	Chlorobenzene	***S*-T(R) [40 µM]**	6.6	12-15
**PS(190)**	Chlorobenzene	***S*-T(R) [40 µM]**	3.7	7
